# Achieving Complex Nanostructures: The Role of Hydrogen in Controlling Mechanical Alloying and Microstructure Evolution in the TiVZrNbHf‐Cu System

**DOI:** 10.1002/advs.202507168

**Published:** 2025-06-30

**Authors:** Lukas Schweiger, Florian Spieckermann, Peter Cengeri, Michael Burtscher, Lukas Schretter, Matthias Eichinger, Gregor Mori, Alexander Schökel, Michael Zehetbauer, Erhard Schafler, Daniel Kiener, Jürgen Eckert

**Affiliations:** ^1^ Department of Materials Science Montanuniversität Leoben Leoben 8700 Austria; ^2^ Faculty of Physics University of Vienna Wien 1090 Austria; ^3^ Erich Schmid Institute of Materials Science Austrian Academy of Sciences Leoben 8700 Austria; ^4^ Chair of General and Analytical Chemistry Montanuniversität Leoben Leoben 8700 Austria; ^5^ Deutsches Elektronen‐Synchrotron DESY 22607 Germany Hamburg

**Keywords:** high entropy alloys, mechanical alloying, metal hydrides, microstructure stabilization, severe plastic deformation

## Abstract

Hydrogen is key in reducing greenhouse gas emissions in materials production. At the same time, it significantly affects mechanical properties, often causing unwanted embrittlement. However, rather than solely addressing these disadvantages, hydrogen's inevitable role in sustainable metallurgy should be leveraged to create new and potentially superior materials. Here, it is shown that using hydrogen in the form of metal hydrides introduces a barrier to mechanical alloying, stabilizing otherwise unattainable microstructures. Severe plastic deformation of a composite of the equiatomic high entropy alloy (HEA) TiVZrNbHf and Cu leads to amorphization, while substituting the HEA with its hydride preserves the two‐phase structure. Monte Carlo simulations confirm that the significantly different hydrogen affinities, together with the restricted dislocation motion in the hydride, create a barrier to mechanical alloying. This hydride route could enable new microstructure states, even in well‐studied material systems. It opens an additional dimension in designing materials containing phases with diverging hydrogen affinities, offering tighter control over mechanical alloying.

## Graphical abstract

This study highlights the drastic influence hydrogen has on the microstructure evolution during severe plastic deformation. High‐pressure torsion of a TiVZrNbHf‐Cu composite results in mechanical alloying and subsequent amorphization, whereas replacing the high entropy alloy with the corresponding hydride stabilizes a two‐phase microstructure. The hydrogen‐mediated inhibition of mechanical alloying offers a new route for preparing other unattainable multi‐phase materials.

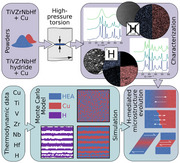

## 1 Introduction

High entropy alloys (HEAs) are intensely investigated for mechanical and functional properties.^[^ ^1^, ^2^ ^]^ In particular, hydride‐forming HEAs are promising candidates for solid‐state hydrogen storage due to the potential for tailoring thermodynamic properties for specific applications.^[^ ^3^ ^]^

Sahlberg et al. suggested that in the equiatomic HEA TiVZrNbHf, due to the high lattice strain introduced by the differences in atomic radii, otherwise inaccessible interstitial sites become available to hydrogen, boosting the hydrogen storage capacity of the material.^[^ ^4^ ^]^ Although subsequent investigations did not confirm such a lattice strain effect,^[^ ^5^ ^]^ TiVZrNbHf and similar variants remain among the best‐researched HEA systems, particularly for hydrogen storage.^[^ ^5^, ^6^, ^7^, ^8^, ^9^ ^]^ Therefore, this system was also chosen as a starting material in this investigation.

Besides developing new alloys, thermomechanical processing is also investigated to pave the way for further property improvements of HEAs:^[^ ^10^, ^11^, ^12^ ^]^ This includes severe plastic deformation (SPD) methods, such as high‐pressure torsion (HPT), which result in nanocrystalline HEAs with improved hydrogen sorption properties^[^ ^13^ ^]^ or superplasticity.^[^ ^11^ ^]^ HPT can also be used for the preparation of (nano)composites^[^ ^14^ ^]^ or supersaturated solid solutions,^[^ ^15^ ^]^ such as W‐Cu,^[^ ^16^ ^]^ FeTi‐Cu,^[^ ^17^, ^18^ ^]^ and Fe‐Cr.^[^ ^19^ ^]^

In this context, it is essential to note that thermomechanical processing can not only enhance hydrogen sorption properties but vice versa, hydrogen and hydrides can significantly impact the plasticity and recovery/recrystallization of alloys.^[^ ^20^, ^21^ ^]^ For example, enhanced strength and ductility were reported for MnCrFeCoNi HEAs due to the impact of hydrogen on the stacking fault energy, enabling nano‐twinning.^[^ ^20^ ^]^ Another work reported accelerated grain growth induced by hydrogen in single‐phase vanadium.^[^ ^21^ ^]^ In contrast to these results, the authors have shown in a previous work that although HPT‐deformed TiVZrNbHf is unstable under high‐temperature conditions, hydrogen tends to stabilize its nanocrystalline structure.^[^ ^22^ ^]^ Such a stabilization could also be associated with hydrogen‐assisted spinodal decomposition, as recently observed by Ma et al.^[^ ^23^ ^]^ This highlights that hydrogen can impact the microstructure and phase evolution of materials in manifold ways.

Consequently, hydrogen might be used as a temporary alloying element,^[^ ^24^ ^]^ such as in Ti alloys during thermo‐hydrogen processing,^[^ ^25^ ^]^ or for producing high‐damping shape‐memory alloys^[^ ^26^ ^]^ and hetero‐structured materials by nano‐twin gradients.^[^ ^27^ ^]^ Further, with the projected widespread use of hydrogen for producing sustainable alloys, e.g., green steels,^[^ ^28^, ^29^ ^]^ such processing routes will become more viable or even unavoidable.^[^ ^30^ ^]^ Although hydrides are known for their brittle behavior,^[^ ^31^ ^]^ recent examples highlight that hydrides might enable overcoming strength‐ductility trade‐offs.^[^ ^32^ ^]^ Beyond that, hydrides are particularly appealing due to their functional properties, such as superconductivity^[^ ^33^ ^]^ and hydrogen storage.^[^ ^34^ ^]^

In this study, we push the concept of using hydrogen to tailor microstructures to the limits by applying it to composites of phases with widely diverging hydrogen affinities. Cu is employed as a second phase due to its excellent deformability,^[^ ^35^ ^]^ high thermal conductivity,^[^ ^35^ ^]^ capacity for selective dissolution,^[^ ^36^ ^]^ and low hydrogen affinity.^[^ ^37^, ^38^ ^]^ Regarding the latter, its incorporation via mechanical alloying can potentially lower the heat of hydride formation, thus destabilizing hydrides and lowering the required desorption temperatures.^[^ ^37^ ^]^ Therefore, powders of the TiVZrNbHf HEA or the respective TiVZrNbHfH_≈10_ HEA hydride are blended with Cu powder and subjected to HPT. Changing from metal to hydride leads to a drastically different microstructure evolution during plastic deformation. For the metal, a metallic glass is formed by mechanical alloying, while for the metal hydride, a two‐phase nanocomposite is stabilized. The prospect of using hydrogen to inhibit mechanical alloying and create otherwise unattainable composite structures is highly appealing. It would be widely applicable to different alloy systems, particularly in view of the anticipated widespread use of hydrogen in future metal and materials production.

## 2 Results and Discussion

**Figure 1:**
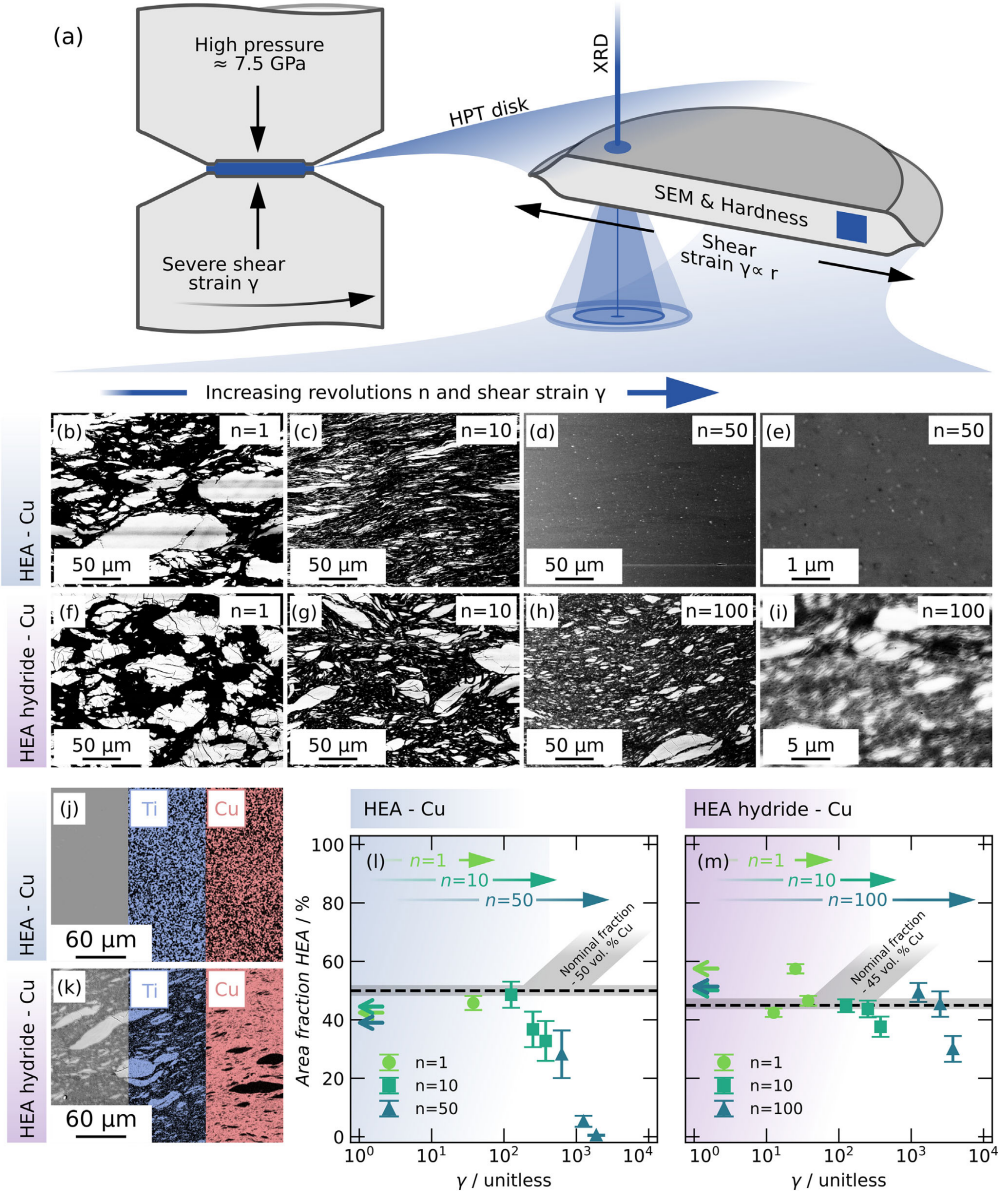
a) Illustration of the HPT process and resulting disks with a radius‐dependent shear strain *γ*. BSE SEM micrographs of HEA‐Cu composites after HPT at RT and b) *n* = 1 (*γ *≈ 34), c) *n* = 10 (*γ* ≈ 428) and d,e) *n* = 50 (*γ *≈ 2142) and of HEA hydride‐Cu composites after HPT at RT and f) *n* = 1 (*γ *≈ 42), g) *n* = 10 (*γ *≈ 471) and h,i) *n* = 100 (*γ *≈ 4712). EDX maps at *n* = 50 and 100 of the j) HEA‐Cu and k) HEA hydride‐Cu composites, respectively. All shown micrographs were taken at a radius of 3 mm. l) HEA and m) HEA hydride phase fractions were determined by image segmentation from the SEM micrographs. Arrows indicate the results at *r *≈ 0 mm and therefore *γ* ≈ 0.

**Figure** **1** shows backscatter electron (BSE) SEM micrographs of the TiVZrNbHf‐Cu (HEA‐Cu) and TiVZrNbHfH_≈10_‐Cu (HEA hydride‐Cu) composites containing 53wt.% Cu, with all micrographs taken at a radius of 3 mm. The Cu phase appears black, and the HEA and HEA hydride are bright. Additionally, the hydride samples exhibit some charging during SEM, likely due to the reduced electrical conductivity of the hydride.

Both composites exhibit refinement of the HEA and HEA hydride phases, respectively. Nevertheless, as seen in Figure 1b–i, differences in the microstructure evolution are visible already at low strains and become more apparent at higher strains.

At *n* = 10 (*γ *≈ 428/471), the HEA phase was drastically elongated, while the hydride deformed to a smaller degree. The refinement appears to be more efficient in the HEA‐Cu composite without hydrogen, and at *n* = 50 (*γ *≈ 2142), the HEA‐Cu composite exhibits a uniform microstructure. No distinction between individual phases was possible, indicating that a nanocrystalline composite or a solid solution formed. Contrarily, the HEA hydride‐Cu composite consists of two well‐distinguishable but significantly refined phases with particle sizes mostly ≤ 1 µm. This is also confirmed by EDX maps in Figure 1j,k, which clearly show the retained chemical partitioning in the hydride composite. Image segmentation results in Figure 1l,m highlight the lower fraction of distinct HEA phase compared to the HEA hydride, the former being hardly resolvable at *n* = 50 (*γ *≈ 2142). A more detailed evaluation, including EDX maps, is given in the Figure S1 and Tables S1 and S2 (Supporting Information).

The evolution of the mechanical properties during HPT can be followed (ex situ) by measuring the radius‐dependent hardness of the composite after HPT and by constructing flow curves from the torque recorded (in situ) during HPT deformation. Beyond basic mechanical properties, the former offers insight into the refinement of the composite, while the latter provides an impression of the deformation behavior of the material during HPT.

**Figure 2:**
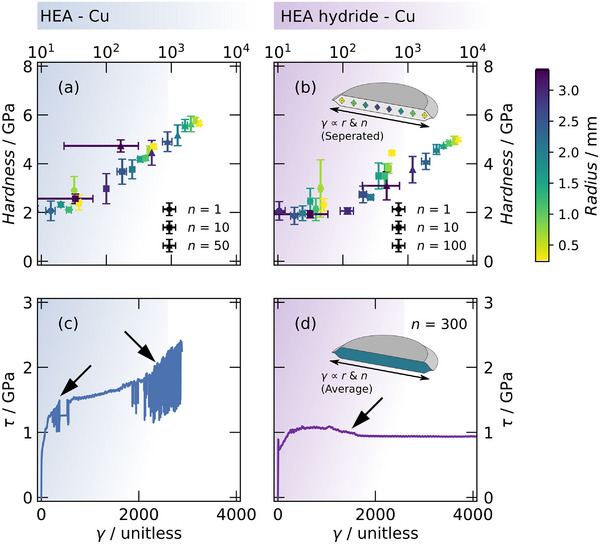
The hardness is plotted as a function of the introduced shear strain *γ* for a) the HEA‐Cu and b) the HEA hydride‐Cu composites. Flow curves constructed from torque measurements, which give the shear stress *τ* as a function of shear strain γ, are shown in c) for the HEA‐Cu and d) for the HEA hydride‐Cu composite.

The hardness and flow curves of the HEA‐Cu composite are plotted in **Figure** **2**a,c. The hardness starts at the same value as for the hydride composite (Figure 2b,d) and increases with HPT deformation, reaching ≈6 GPa without any observable saturation plateau.

In line with this, the HEA‐Cu composite also exhibits a continuous increase in shear stress, indicating ongoing hardening without any signs of strain localization. One reason for this might lie in the better deformability of the metallic bcc HEA compared to the corresponding hydride. The significant hardening of the HEA‐Cu composite limits further deformation as plastic instabilities or slip‐stick events, indicated by arrows in Figure 2c, occur at higher numbers of revolutions.^[^ ^39^ ^]^ In the latter case, the contact friction between the anvil and sample becomes too weak to facilitate further HPT processing and refinement.

The hardness of the HEA hydride‐Cu composite is plotted against the introduced strain in Figure 2b, revealing an increase in hardness from ≈ 2 to ≈ 5 GPa and a plateau at higher strains.

The complementary Figure 2d depicts the flow curve of the HEA hydride‐Cu composite. The curve exhibits an initial rise in shear stress *τ*, followed by a slight reduction indicated by an arrow and presumably associated with the onset of strain localization in the softer composite phase, i.e., in Cu. Such strain localization significantly imparts further refinement of the HEA hydride phase, as observed, for example, in the Mg‐Fe system.^[^ ^40^ ^]^ Higher resolution micrographs in Figure 1 show limited refinement; no homogenous nanocomposite, as for the W‐Cu^[^ ^36^, ^41^ ^]^ or FeTi‐Cu^[^ ^17^, ^18^ ^]^ systems, was obtained. Increasing the number of revolutions further to *n* = 300 results, not in further refinement but severe cracking.

These differences in the deformation behavior of both phases align with the SEM micrographs in Figure 1, with the HEA particles exhibiting significantly more elongation and necking than the respective hydride particles. Additionally, in the HEA‐Cu composite, potential localization events might be suppressed by mechanical alloying and the resulting solid solution strengthening.

**Figure 3:**
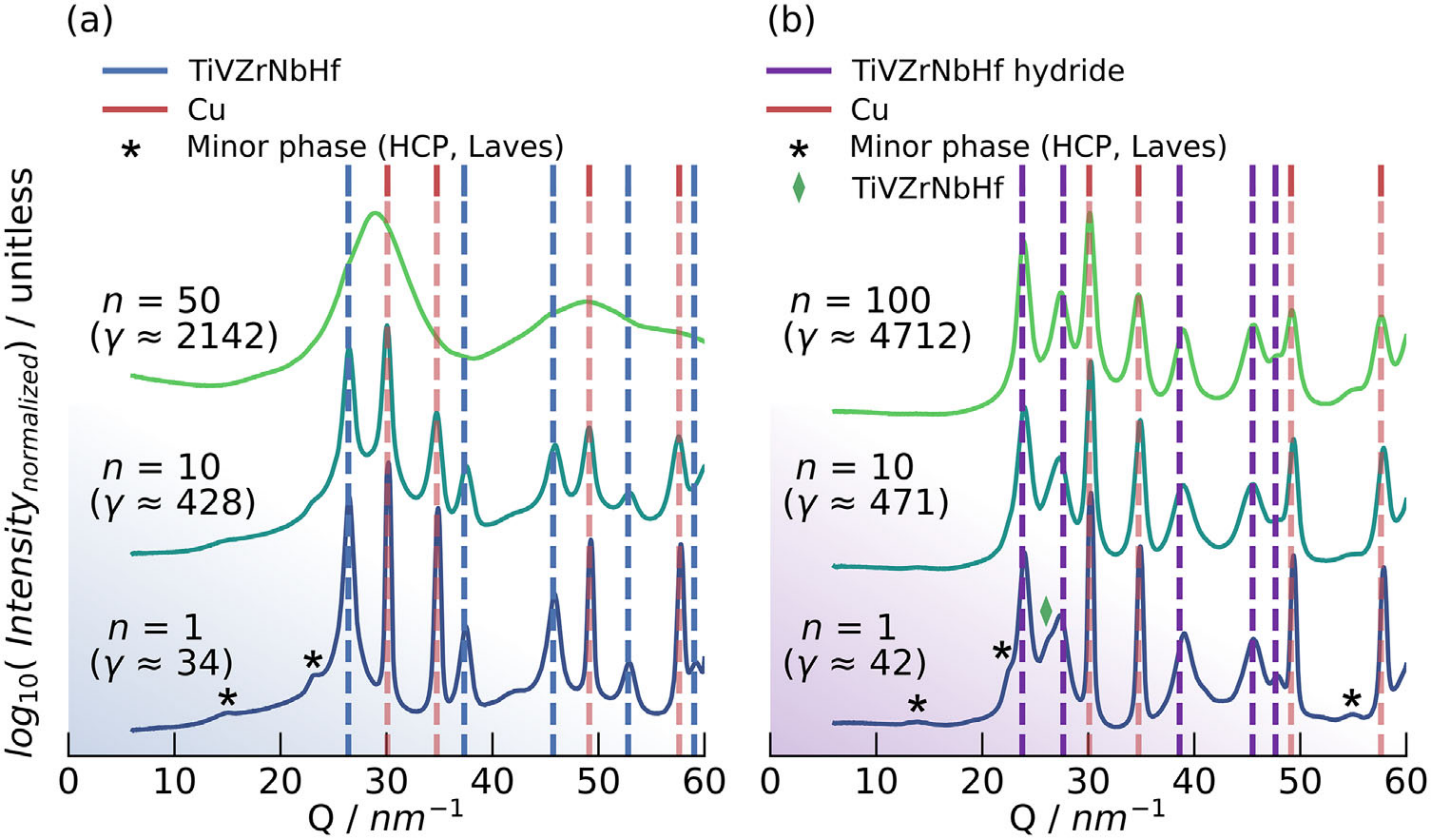
Synchrotron XRD patterns of a) the HEA‐Cu and b) the HEA hydride‐Cu composites at *n* = 1, 10, 50, and 100, respectively. The beam was centered at *r* ≈ 3mm. The corresponding shear strains *γ* at these radii are given.

The significant structural differences after HPT‐deformation are confirmed by synchrotron X‐ray diffraction (XRD) measurements in **Figure** **3**. Both HEA‐Cu and HEA hydride‐Cu composites initially consist of a distinct two‐phase microstructure. At higher strains, the HEA‐Cu composites transform into a single‐phase material and exhibit (partial) amorphization, as highlighted by the broad diffraction maxima in the XRD patterns. A minor shoulder associated with the HEA is still visible, highlighting the incomplete amorphization. Further XRD patterns plotted in Figure S2 (Supporting Information) and recorded as a function of radius show that amorphization only occurs at the largest radii due to the largest strains being present here. In fact, the HEA‐Cu composite includes elements like Ti, Zr, and Cu, which are known to promote amorphization so that partial amorphization can be expected.^[^ ^42^ ^]^ Studies on Ti‐(Zr)‐Cu also found partial amorphization during HPT.^[^ ^43^, ^44^ ^]^

In agreement with the microstructure determined by SEM and irrespective of strain, the HEA hydride‐Cu composite retains the two‐phase structure, with X‐ray peak broadening due to the grain refinement and dislocation accumulation in both phases during HPT deformation. This indicates that hydrogen, in the form of the respective hydride, significantly restricts mechanical alloying and subsequent solid‐state amorphization.

**Figure 4:**
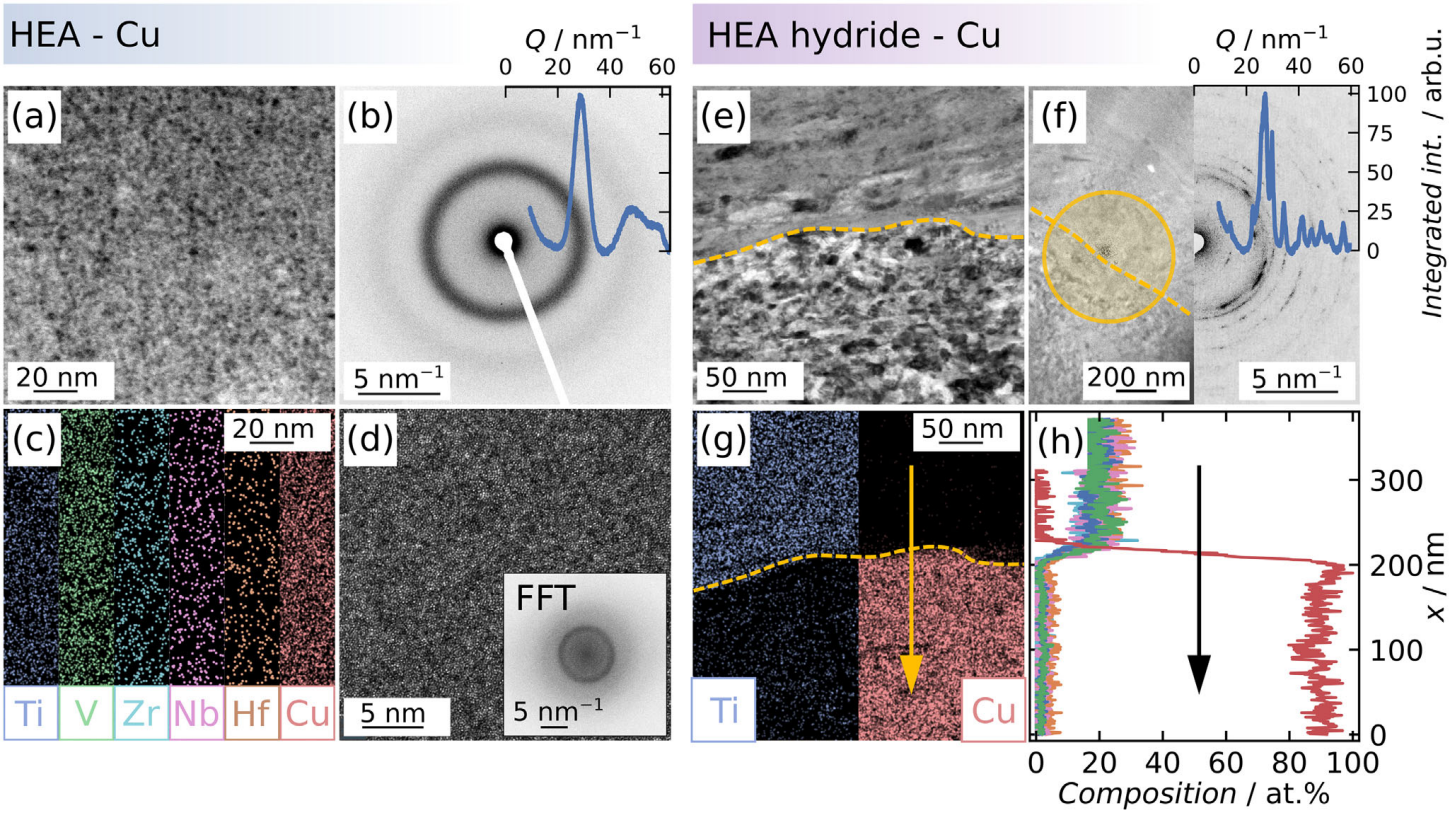
a) STEM micrographs of the HEA‐Cu composite (*r *> 3 mm, *n* = 50) and the associated b) SAED pattern, c) EDX map, and d) HRTEM micrograph. The latter includes an inset with the associated Fast Fourier Transformation (FFT). e) STEM micrographs of the HEA hydride‐Cu composite (*r *> 3 mm, *n* = 100), the associated f) SAED pattern, g) EDX map, and h) derived line scan.

The XRD results are complemented by locally probing the microstructure by (S)TEM, with the results of the HEA‐Cu and HEA hydride‐Cu composites provided in **Figure** **4**. In the case of the HEA‐Cu composite, slight structural heterogeneities are visible in the STEM micrographs in Figure 4a, but the SAED pattern and the high‐resolution micrograph in Figure 4b,d confirm the amorphous nature of the severely deformed material. The results also align with the XRD results given above. As seen in the EDX maps in Figure 4c, the amorphous metal exhibits a high degree of chemical homogeneity. Therefore, this nanoscale investigation indicates pronounced HPT‐induced mechanical alloying and consequent solid‐state amorphization.

In contrast, the HEA hydride‐Cu composite had a distinct two‐phase microstructure, with Figure 4e depicting the corresponding STEM micrograph. Both phases display significant refinement and a highly defective structure. The SAED pattern and EDX map in Figure 4f,g highlight the crystalline nature of both phases as well as a distinct and persistent chemical separation. An associated line scan over the HEA hydride/Cu interface plotted in Figure 4h highlights the sharp chemical differences with no significant interdiffusion. In the HEA hydride phase, minor segregation of V is visible, which is in line with the reported preferred grain boundary segregation of V in this system.^[^ ^45^ ^]^ Consequently, although the HEA hydride‐Cu composite exhibits significant refinement and defect accumulation due to the severe plastic deformation, the nanoscale morphology indicates a significant barrier against interdiffusion and mechanical alloying at the hydride/Cu interface.

The structural investigation was supplemented by differential scanning calorimetry (DSC) to determine potential stability differences between the respective composites. The measurements revealed distinct exothermic crystallization peaks in the case of the (partially) amorphous HEA‐Cu composites. The most severely deformed HEA hydride‐Cu composite also decomposes exothermically, presumably following and thereby masking the expected endothermic dehydrogenation. These results, detailed in Figures S3–S5 (Supporting Information), suggest an important role of hydrogen in stabilizing the otherwise unstable two‐phase microstructure. The data indicates that the thermal stability of the HEA hydride‐Cu composite is governed by the hydride desorption, with the subsequent decomposition starting at 550 °C. In contrast, the amorphous HEA–Cu composite decomposes at a lower temperature of ≈430 °C and subsequently at 580 °C.

The results show that replacing the metal with its corresponding hydride effectively inhibits mechanical alloying. Mechanical alloying occurs via mechanical mixing and diffusion‐assisted mechanisms.^[^ ^15^ ^]^ Mechanical mixing models, such as kinetic roughening^[^ ^46^ ^]^ and dislocation shuffling,^[^ ^47^ ^]^ involve continuous shearing of the respective phases, irrespective of mixing enthalpies. Pairings with unlikely mechanical properties, such as HEA hydride‐Cu, tend to follow an erosion and abrasion model, leading to gradual refinement and subsequent mechanical mixing.^[^ ^18^, ^48^, ^49^ ^]^

Diffusion can aid or hinder alloying, depending on the mixing enthalpy.^[^ ^50^ ^]^ However, even for a positive enthalpy of mixing, high defect and phase boundary densities can drive diffusion‐assisted dissolution, with small particles dissolving due to the Gibbs–Thomson effect.^[^ ^47^ ^]^

These mechanisms are not necessarily exclusive but can work sequentially, with initial mechanical refinement, e.g., by dislocation shuffling, enabling subsequent atomic‐scale dissolution by diffusion.^[^ ^47^ ^]^ Consequently, the observed suppression of mechanical alloying could originate from both limited diffusion and/or mechanical mixing.

Regarding diffusion, the impeded mechanical alloying and related composite stabilization can be rationalized by the different affinities of the respective composite phases for hydrogen. The HEA, consisting of elements with high hydrogen affinity, has negative enthalpies of hydrogen dissolution and hydride formation.^[^ ^6^ ^]^ For metallic Cu, in contrast, the enthalpy of dissolution is positive.^[^ ^37^, ^51^ ^]^ The corresponding values are given in **Table** **1**. Consequently, we hypothesize that the diverging structural evolution is rooted in the different hydrogen affinities and associated thermodynamics, particularly in the later stages of HPT. Therefore, the thermodynamics of the HEA(H)‐Cu system are outlined below. For details, the reader is referred to Tables S3 and S4 and Equations S1–S4 (Supporting Information).

**Table 1:** Enthalpies of hydrogen dissolution Δ*H*_diss,_ and enthalpies of hydride formation Δ*H*_hydride_ of Cu and the HEA elements.^[^ ^6^, ^37^, ^51^ ^]^

	Cu^[^ ^37^, ^51^ ^]^	Ti^[^ ^37^, ^51^ ^]^	V^[^ ^37^, ^51^ ^]^	Zr^[^ ^37^, ^51^ ^]^	Nb^[^ ^37^, ^51^ ^]^	Hf^[^ ^37^, ^51^ ^]^	TiVZrNbHf^[^ ^6^ ^]^
Δ*H*_diss_ / kJ mol^−1^ H	+ 43–55	− 52 (α‐Ti)	− 26–33	−52‐64 (α ‐Zr)	−33‐38	−38	n.a.
Δ*H*_hydride_ / kJ mol^−1^ H	n.a.	−68 (TiH_2_)	−36‐42 (VH_0.5_)	−82‐106 (ZrH_2_)	−38 (NbH_0.5_)	−66 (HfH_2_)	−30 (MH_1.9‐2.5_)

Based on XRD and TEM, HPT of the HEA‐Cu composite results in complete dissolution of the constituent HEA atoms, i.e., Ti, V, Zr, Nb, Hf, in the Cu phase and vice versa. As seen in Table S3 (Supporting Information), Ti, Zr, and Hf have high negative mixing enthalpies with Cu,^[^ ^52^ ^]^ and their respective phase diagrams show the formation of intermetallics.^[^ ^53^ ^]^ V and Nb, however, show low but positive mixing enthalpies.^[^ ^52^ ^]^ Nevertheless, all individual elements exhibit some degree of dissolution in Cu when subjected to, e.g., ball milling.^[^ ^54^, ^55^, ^56^, ^57^, ^58^, ^59^ ^]^ For a more quantitative picture of the complex system, the enthalpies and entropies of mixing, Δ*H*_mix_ and Δ*S*_mix_, for a Cu‐TiVZrNbHf phase were derived according to the procedure proposed by Yang and Zhang,^[^ ^60^ ^]^ using thermodynamic data from Takeuchi and Inoue.^[^ ^52^ ^]^ The resultant free enthalpy Δ*G*_mix_ is found as −10.2 kJ mol^−1^, and consequently, a single‐phase solid‐solution, either crystalline or amorphous, is expected, as confirmed experimentally.

**Figure 5:**
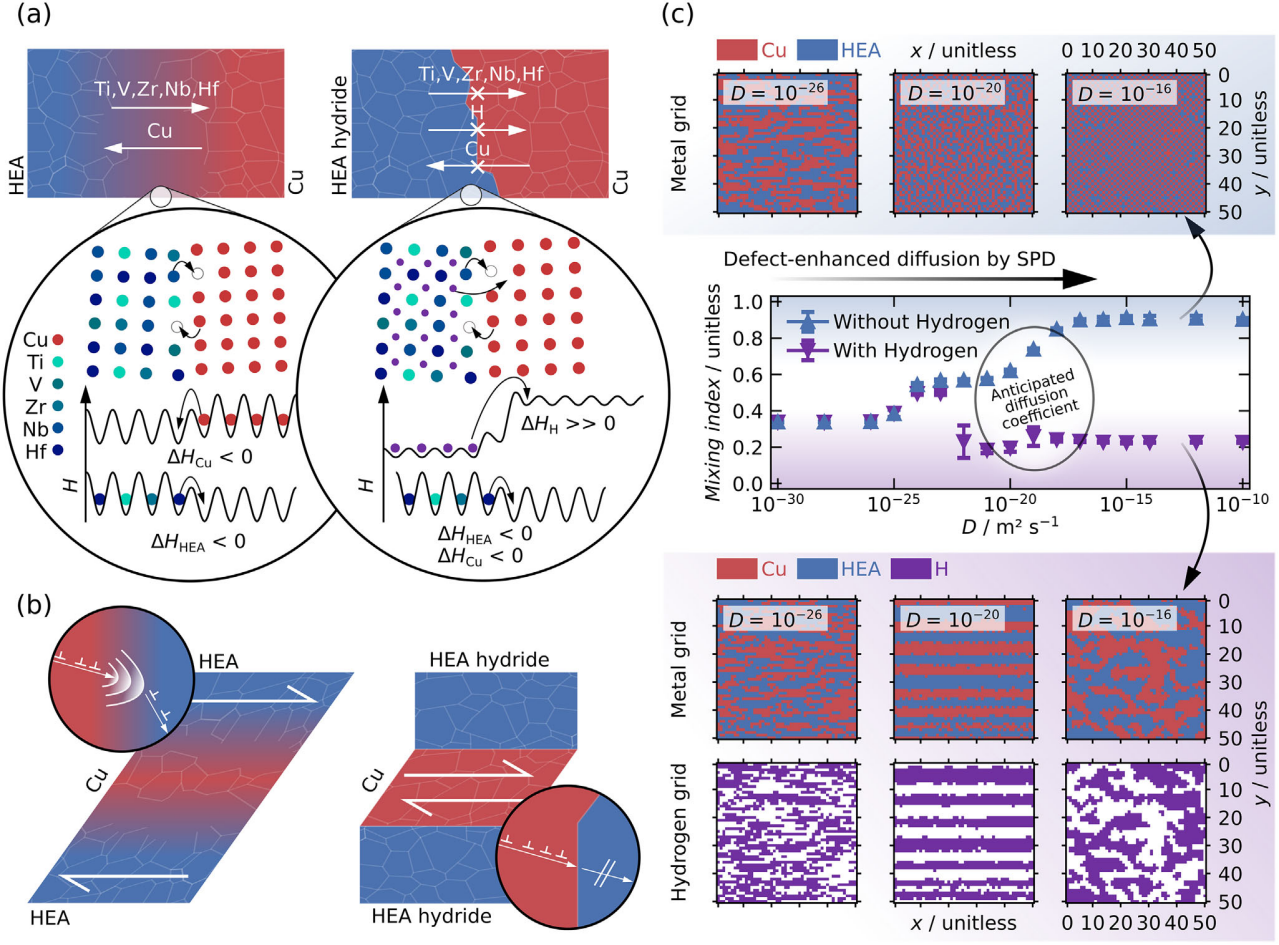
Illustration of the proposed mechanisms impeding a) mechanical alloying and b) co‐deformation during deformation of the HEA‐Cu and HEA hydride‐Cu composites. c) Mixing indices derived from the Monte Carlo simulations, assuming different diffusion coefficients and including exemplary metal and hydrogen grids.

However, in the case of the hydride, hydrogen must also be accommodated during mechanical alloying. Therefore, we suggest that metal and hydrogen dissolution are coupled, implying that hydrogen released from the HEA hydride would need to dissolve into the Cu phase simultaneously. Assuming a hydrogen‐to‐metal ratio of two, two hydrogen atoms would be dissolved in Cu for every HEA atom.^[^ ^6^ ^]^ The free enthalpy Δ*G*_mix_ for this process was derived by considering Δ*H*_diss_ and Δ*H*_hydride_ for Cu and TiVZrNbHf in Table 1, as well as the entropy gain by spreading hydrogen over both phases, yielding a Δ*G*_mix_‐value of +21.5 or +26.4 kJ mol^−1^, assuming a bcc or fcc solid solution, respectively. Such coupling of metal and hydrogen diffusion would impart a significant positive enthalpy on the whole process, and the free enthalpy of mixing overall becomes >> 0. Consequently, the formation of a single‐phase solid solution and subsequent amorphization become thermodynamically blocked: Diffusion will counteract rather than promote mechanical mixing. This proposed mechanism is illustrated in **Figure** **5**a.

The second critical factor is mechanical mixing itself, which is tightly linked to the required flow stresses, plastic deformation behavior, and the associated fragmentation of the constituent phases. In the HEA‐Cu composite, the anticipated formation of an amorphous phase boundary between Cu and HEA should promote more uniform stress transmission across the interface, facilitating homogenous dislocation nucleation and subsequent co‐deformation and mechanical mixing.^[^ ^61^, ^62^ ^]^

In contrast, metal hydrides are generally considered brittle compared to their metallic counterparts. Limited plasticity and dislocation motion are possible in compression but are severely limited.^[^ ^31^ ^]^ At the same time, cross‐slip is also highly restricted.^[^ ^31^ ^]^ Dislocation shuffling,^[^ ^47^ ^]^ i.e., the propagation of dislocations through phase boundaries, could thereby be prevented due to the change in crystal structure from bcc to distorted fcc^[^ ^6^ ^]^ or the overall lowered dislocation mobility. Therefore, such material pairing will likely inhibit co‐deformation and mechanical mixing but promote deformation localization. Based on the flow curves in Figure 2, the latter was observed in the HEA hydride‐Cu but not the HEA‐Cu composite. As illustrated in Figure 5b, this localization tendency could be additionally promoted by the absence of mechanical alloying and associated solid solution strengthening.

To support these mechanistic considerations, Monte Carlo (MC) simulations were performed to mimic and conceptualize the microstructure evolution, using the enthalpies of mixing Δ*H*_mix_ estimated above as input parameters. Metal–metal composites and metal hydride‐metal composites were modeled, including metal diffusion, hydrogen diffusion, and shear steps representing HPT‐deformation. The latter was realized by translating one part of the grid by one site along a randomly chosen vertical or horizontal line. The respective probabilities are based on the experimental strain rates and assumed diffusion coefficients.

The results are summarized in Figure 5c, and a more complete overview of the assumptions and results is provided in the Supporting Information, including Figures S6–S13 (Supporting Information).

It should be noted that MC simulations cannot capture the complex defect structure present during HPT deformation. However, such defects can impact diffusion, plastic deformation, and, to some extent, the mixing enthalpies, e.g., by increased hydrogen solubilities at defects.

Therefore, to account for potential variations of the respective MC inputs, a Morris sensitivity analysis is used to confirm the stability of the simulation results.^[^ ^63^ ^]^ The diffusion coefficient had the greatest impact on the results, followed by the A‐B, A‐H, and B‐H interaction energies with moderate to minor influence. Given that the diffusion coefficient was varied across several orders of magnitude, we conclude that the MC simulation results remain robust despite the defect‐related uncertainties in the input parameters. The detailed results are given in the Tables S5 and S6 (Supporting Information).

As mentioned above, the diffusion coefficient is crucial in setting the diffusion and shear probabilities during each MC step. Therefore, the diffusion coefficient was varied from 10^−10^ to 10^−30^ m^2^ s^−1^, as this input parameter needed to be estimated, and as studies suggest significantly increased diffusion coefficients during SPD due to high defect densities.^[^ ^64^, ^65^ ^]^ The lower bound represents unaffected bulk diffusion, while the upper bound represents rapid diffusion. Opposed to this, the strain rate could be derived directly from the HPT settings.

Comparing the MC results in Figure 5c, one can see that despite the unchanged metal–metal interactions, the significant differences in the metal–hydrogen interactions result in an entirely different microstructure evolution. At diffusion coefficients above ≈ 20^−23^ m^2^ s^−1^, the HEA‐Cu system evolved toward a mixed state, while the HEA hydride‐Cu systems stayed well separated. The results of the metal and metal hydride composites only converge at lower diffusion coefficients. Under such circumstances, mechanical mixing by shear, which neglects any associated energy changes, starts to dominate, and diffusion occurs less frequently. At diffusion coefficients below 10^−25^ m^2^ s^−1^ the mixing index decreased further. Under these conditions, diffusion is absent, and any mixing is solely mediated by shear. This indicates that mechanical mixing is insufficient for forming a (supersaturated) solid‐solution, but diffusional processes are still required eventually.

However, it is well investigated that SPD‐processed materials,^[^ ^66^ ^]^ as well as nanocrystalline/metastable materials^[^ ^67^ ^]^ in general, exhibit significantly enhanced diffusion. Relevant studies that determined diffusion coefficients have been collected and are summarized in Table S7 (Supporting Information).^[^ ^64^, ^65^, ^68^, ^69^, ^70^, ^71^, ^72^, ^73^, ^74^, ^75^, ^76^, ^77^, ^78^, ^79^ ^]^ Estimations of the lattice, grain boundary, and surface diffusivities in the TiVZrNbHf‐Cu system are also provided in the Supporting Information, including Figure S14 (Supporting Information). Based on these results, the diffusion coefficient during HPT was anticipated to be in the range between 10^−18^ and 10^−21^ m^2^ s^−1^, in which the MC simulations yield strongly different results for HEA‐Cu and HEA hydride‐Cu composites, respectively.

It should be noted that the MC simulations do not include the restricted dislocation shuffling in the hydride case. Again, detailed results of the MC simulation are provided in the Supporting Information, including Figures S6–S13 (Supporting Information).

As mentioned above, in the case of the HEA‐Cu composite, mechanical alloying and the strengthening of the associated solid solution can potentially reduce differences in flow stress between the respective composite phases. Contrarily, the absence of appreciable mechanical alloying in the hydride composite will render localization or shear band formation more likely. The Monte Carlo simulations confirm these experimental observations. When the probability of the shear steps is scaled by a hypothetical flow stress derived from the chemical composition along the shear line, interdiffusion in the HEA‐Cu composite tends to smooth the localization, as seen in Figure S7 (Supporting Information). In the case of the HEA hydride‐Cu composite, such localization phenomena are more pronounced, see Figure S11 (Supporting Information), even more so in the MC simulations introducing only horizontal shear. It should be noted, however, that incorporating such flow stress effects reproduced certain aspects of the experimental results but did not significantly change the overall simulation results.

Therefore, the MC model confirms the experimental results of the flow curves in Figure 2, highlighting that hydrogen and its subsequent effects can significantly influence material behavior during severe plastic deformation, impacting microstructure evolution and coupled solid‐state amorphization.

Overall, the experimental and MC results align well, offering deeper insights into the connections between thermodynamics, deformation behavior, and resulting microstructure evolution of the system during SPD. Despite this agreement, and although the impact of hydrogen on the microstructure evolution is evident, it is noted that the current explanation of hydrogen‐inhibited mechanical alloying and the associated free‐energy arguments are current hypotheses. In particular, it has at this point not been possible to disentangle the contributions of i) inhibition of mechanical alloying and ii) mechanical effects introduced by the reduced plastic deformability of the hydride compared to the metal. Consequently, further investigations will be required to obtain direct experimental evidence for the proposed mechanism.

Another important consideration is how broadly the proposed processing route and associated mechanism can be applied. This study deliberately probed the extremes of how strongly hydrogen can influence microstructure evolution. A prerequisite for a pronounced effect is a substantial contrast in hydrogen affinity: one phase forms a stable hydride while the other exhibits low hydrogen solubility. A prominent system fulfilling this, which has been investigated extensively in the past, including upscaling strategies, is Nb‐Cu.^[^ ^80^, ^81^, ^82^ ^]^ It might be usable for this proposed new *hydride route*. However, materials such as dual‐phase steels and dual‐phase HEAs, featuring smaller differences in hydrogen affinity, are expected to show weaker effects. Future work should, therefore, map the boundary conditions for using hydrogen to tailor multi‐phase microstructures.

## 3 Conclusion

In this study, we used hydrogen to achieve microstructures otherwise not attainable by SPD. HPT was used to deform a composite composed of the equiatomic HEA TiVZrNbHf and Cu, and due to the unavoidable mechanical alloying and interdiffusion, a (partially) amorphous material formed. However, exchanging the HEA with the corresponding TiVZrNBHfH_≈10_ hydride enables an entirely different microstructure evolution. The proposed reason lies in the significantly different affinities of the constituent phases to hydrogen, with the HEA possessing a high and Cu having a low hydrogen affinity. Because significant mechanical alloying of metal atoms also requires the redistribution of hydrogen, these processes become energetically coupled, and consequently, a sizeable energy barrier against mechanical alloying is introduced. In this work, this mechanism was confirmed by a simplified model based on Monte Carlo simulations, mimicking the chemically complex system. In addition, the absence of mechanical alloying and restricted dislocation mobility in the hydride limits the direct mechanical mixing of the constituent phases.

It is concluded that during HPT − as an exemplary deformation technique − a hydrogen‐induced barrier to mechanical alloying can be introduced in a composite that consists of phases with significantly different hydrogen affinities. Within these boundaries, our investigated approach paves the way for designing and producing complex multi‐phase composites, which are otherwise difficult to obtain but may possess unique functional properties, utilizing hydrogen as a, potentially temporary, alloying element.

## 4 Experimental Section

### Material Preparation

The equiatomic HEA TiVZrNbHf was synthesized by arc melting (Arc Melter AM 0.5, Edmund Bühler GmbH, Germany) stoichiometric amounts of Ti (99.995%, HMW Hauner GmbH, Germany), V (99.9%), Zr (99.2%), Nb (99.9%), and Hf (99.9%). Subsequently, the ingot was exposed to 40 bar of hydrogen at 350 °C, causing self‐pulverization due to the substantial volume expansion (≈ 26%)^[^ ^6^ ^]^ upon hydride formation.

The obtained hydride powder was heated to 500 °C in a vacuum furnace to desorb the hydrogen, yielding the corresponding dehydrogenated HEA powder. XRD patterns before and after annealing, as shown in Figure S15 (Supporting Information), confirm complete desorption. SEM micrographs of the powder are shown in Figure S16 (Supporting Information).

The absorption (1.55 wt.% hydrogen) and complete desorption (16.5 wt. ppm hydrogen) of the powders was confirmed by thermal desorption spectroscopy (TDS, Galileo G8, Bruker, USA; coupled with an IR07 infrared furnace and IPI quadrupole mass spectrometer). The results and respective TDS spectra are provided in the Figure S17 (Supporting Information).

The resultant HEA and respective HEA hydride powders (determined area‐weighted particle sizes of 115 ± 102 and 129 ± 98 µm, respectively) were blended with Cu powder (170 + 400 mesh, 88–38 µm) to obtain composites with 53 wt.% Cu. The powders were mixed using a mortar and pestle in a glove box with O_2_ and H_2_O levels below 0.5 ppm. The composites were subsequently compacted under inert gas conditions as described elsewhere.^[^ ^17^ ^]^ Following, severe plastic deformation was introduced by HPT at room‐temperature (RT), a pressure of 7.5 GPa, and under ambient atmosphere, with anvils having a cavity diameter and depth of 8 and 0.15 mm, respectively. The speed of deformation was set to 1.27 rpm. The anvils were cooled with pressurized air during deformation to avoid excessive heating, and the temperature is expected to remain significantly below the desorption temperature of the HEA hydride. The torque *T* was measured during HPT to determine the torsional shear stress τ present during deformation, which was calculated by

(1) $$\tau =\frac{3T\;}{2\pi r^{3}}\;$$

with *r* being the radius of the HPT disk. The torsional shear strain *γ* was calculated as

(2) $$\gamma =\frac{2\pi nr}{t}\;$$

with *n* being the number of revolutions, *r* the radius at which the strain was calculated, and *t* the thickness of the HPT disk.^[^ ^83^ ^]^ The HPT disks had a diameter and thickness of ≈ 8 and 0.5 mm, respectively.

### Material Characterization

The resultant microstructure was investigated by scanning electron microscopy (SEM, LEO type 1525, Carl Zeiss GmbH, Germany) and energy dispersive X‐ray spectroscopy (EDX; XFlash 6–60, Bruker, USA) on mirror‐polished cross‐sections of the HPT disks. The same cross‐section was subsequently used to determine the radius‐dependent microhardness (DuraScan, ZwickRoell GmbH, Germany). X‐ray diffraction (XRD) measurements were done at the P02.1 Powder Diffraction and Total Scattering Beam Line of PETRA III (DESY Hamburg) in transmission geometry, with a photon energy of 60 keV and a Dectris EIGER2X CdTe 1M‐W detector. The calibration was done using a CeO_2_ calibrant. The HEA and HEA hydride starting powders were characterized using a benchtop X‐ray powder diffractometer using a Co‐K_α_ source (D2 Phaser, Bruker, USA).

Transmission electron microscopy (TEM, JEM‐2200FS microscope, JEOL Ltd., Japan) and EDX maps (Ultim Max TEM window‐less detector, Oxford Instruments plc, UK) were recorded on lamella prepared and ion‐polished using a plasma focused ion beam microscope (PFIB, Helios 5 PFIB CXe, ThermoFischer, USA).

Differential scanning calorimetry (DSC, heat flux differential scanning calorimeter Netzsch DSC 404 F1 Pegasus, Netzsch, Germany) was conducted on quartered HPT disks with the less deformed central region, i.e., *r* < 2 mm, removed.

### Monte Carlo Simulations

Monte Carlo simulations were implemented using Python. The input parameters determining metal and hydrogen diffusion and the shear processes mimicking HPT deformation were based on reported thermodynamic data and the experimental strain rates. Details of the thermodynamic calculations, the simulations, and the Morris sensitivity analysis^[^ ^63^ ^]^ are provided in the Supporting Information.

## Conflict of Interest

The authors declare no conflict of interest.

## Supporting information



Supporting Information

## Data Availability

The data that support the findings of this study are openly available in Experimental data and Monte Carlo simulation results associated with “Achieving Complex Nanostructures: The Role of Hydrogen in Controlling Mechanical Alloying and Microstructure Evolution in the TiVZrNbHf‐Cu System” at https://doi.org/10.5281/zenodo.15746219, reference number 15746219. The Monte Carlo code is available on GitHub (https://github.com/LSchweiger‐Science).
