# Supplementary material for: Achieving Complex Nanostructures: The Role of Hydrogen in Controlling Mechanical Alloying and Microstructure Evolution in the TiVZrNbHf‐Cu System
Source: Adv Sci (Weinh). 2025 Jun 30;12(33):e07168. doi: 10.1002/advs.202507168 (PMC12412505; doi:10.1002/advs.202507168)
Supplement: Supplementary file 1 — Supporting Information [file ADVS-12-e07168-s001.docx]

Supporting Information

**Achieving Complex Nanostructures: The Role of Hydrogen in Controlling Mechanical Alloying and Microstructure Evolution in the TiVZrNbHf-Cu System**

*Lukas Schweiger*^1^, Florian Spieckermann*^1^, Peter Cengeri^2^, Michael Burtscher^1^,
Lukas Schretter^3^, Matthias Eichinger^4^, Gregor Mori^4^, Alexander Schökel^5^,
Michael Zehetbauer^2^, Erhard Schafler^2^, Daniel Kiener^1^, Jürgen Eckert^1,3^*

1 Department of Materials Science, Montanuniversität Leoben, Leoben, 8700, Austria

2 Faculty of Physics, University of Vienna, Wien, 1090, Austria

3 Erich Schmid Institute of Materials Science, Austrian Academy of Sciences, Leoben, 8700, Austria

4 Chair of General and Analytical Chemistry, Montanuniversität Leoben, Leoben, 8700, Austria

5 Deutsches Elektronen-Synchrotron DESY, Hamburg, 22607, Germany

***E-mail: lukas.schweiger@unileoben.ac.at, florian.spieckermann@unileoben.ac.at

1. EDX characterization

**Table S1:** Chemical compositions of the HEA-Cu composite obtained from EDX measurements of the initial (compacted) microstructure, as-HPT deformed microstructure (RT, n = 50, see **Figure S1**), and following DSC analysis at temperatures up to 1000 °C. The phases after DSC are indicated in **Figure S4**.

|  | **Ti / at. %** | **V / at. %** | **Cu / at. %** | **Zr / at. %** | **Nb / at. %** | **Hf / at. %** |
| --- | --- | --- | --- | --- | --- | --- |
| **HEA - Cu - RT - n = 1 - 0 mm (*γ* ≈ 0, initial microstructure)** | | | | | | |
| HEA | 22.2 ± 0.5 | 19.6 ± 0.5 | 1.1 ± 0.6 | 19.7 ± 0.5 | 19.7 ± 0.7 | 17.7 ± 0.5 |
| Cu | 0.0 ± 0.0 | 0.0 ± 0.0 | 99.4 ± 0.2 | 0.0 ± 0.0 | 0.0 ± 0.0 | 0.4 ± 0.1 |
| **HEA - Cu - RT - n = 50 - 3 mm (*γ* ≈ 2142, as-HPT)** | | | | | | |
| HEA-Cu glass | 8.9 ± 0.5 | 8.0 ± 0.5 | 60.0 ± 2.5 | 8.0 ± 0.6 | 8.1 ± 0.6 | 7.0 ± 0.5 |
| **HEA hydride - Cu composite - RT- n = 50 - Post-DSC (to 1000 °C, 10 K s^−1^)** | | | | | | |
| Phase 1 (Large grains) | 4.7 ± 1.2 | 0.2 ± 0.1 | 76.0 ± 0.4 | 10.9 ± 0.8 | 0.3 ± 0.1 | 7.9 ± 0.5 |
| Phase 2 (Globular grains) | 8.0 ± 0.9 | 40.1 ± 1.9 | 6.5 ± 4.0 | 0.1 ± 0.2 | 44.9 ± 2.9 | 0.3 ± 0.3 |
| Phase 3 (Intergranular) | 7.4 ± 3.2 | 0.3 ± 0.1 | 91.4 ± 3.4 | 0.1 ± 0.2 | 0.0 ± 0.0 | 0.9 ± 0.4 |
| Phase 4  (Interphase) | 19.1 ± 3.5 | 0.7 ± 0.7 | 76.2 ± 4.7 | 1.2 ± 1.2 | 0.5 ± 0.6 | 2.3 ± 2.4 |

**Table S2:** Chemical compositions of the HEA hydride-Cu composite obtained from EDX measurements of the initial (compacted) microstructure, as-HPT deformed microstructure (RT, n = 100, see **Figure S1**), and following DSC analysis at temperatures up to 1000 °C. The phases after DSC are indicated in **Figure S5**.

|  | **Ti / at. %** | **V / at. %** | **Cu / at. %** | **Zr / at. %** | **Nb / at. %** | **Hf / at. %** |
| --- | --- | --- | --- | --- | --- | --- |
| **HEA hydride - Cu composite - RT- n = 1 - 0 mm (*γ* ≈ 0, initial microstructure)** | | | | | | |
| HEA hydride | 22.2±0.5 | 20.2±1.7 | 1.1±0.5 | 19.5±0.8 | 19.7±1.7 | 17.3±0.7 |
| Cu | 0.0±0.0 | 0.1±0.0 | 99.4±0.2 | 0.0±0.0 | 0.0±0.0 | 0.5±0.2 |
| **HEA hydride - Cu composite - RT- n = 100 - 3 mm (*γ* ≈ 4712, as-HPT)** | | | | | | |
| HEA hydride | 21.9±0.4 | 19.4±0.8 | 2.5±0.7 | 19.6±0.5 | 19.7±0.6 | 16.9±0.4 |
| (Nano)composite | 6.4±1.6 | 5.7±1.4 | 71.5±6.7 | 5.5±1.3 | 5.7±1.4 | 5.2±1.1 |
| **HEA hydride - Cu composite - RT- n = 100 - Post-DSC (to 1000 °C, 20 K s^−1^)** | | | | | | |
| Phase 1 (Large grains) | 4.9±1.9 | 0.1±0.1 | 76.1±0.6 | 11.8±0.8 | 0.3±0.1 | 6.7±0.9 |
| Phase 2 (Globular grains) | 7.4±0.9 | 42.4±0.8 | 3.8±0.3 | 0.0±0.1 | 46.3±0.8 | 0.1±0.1 |
| Phase 3 (Intergranular) | 8.0±1.0 | 0.3±0.1 | 91.1±1.1 | 0.0±0.0 | 0.0±0.0 | 0.5±0.1 |
| Phase 4  (Interphase) | 18.9±4.7 | 1.1±1.0 | 75.8±5.5 | 1.3±0.8 | 1.2±1.3 | 1.7±0.8 |

**
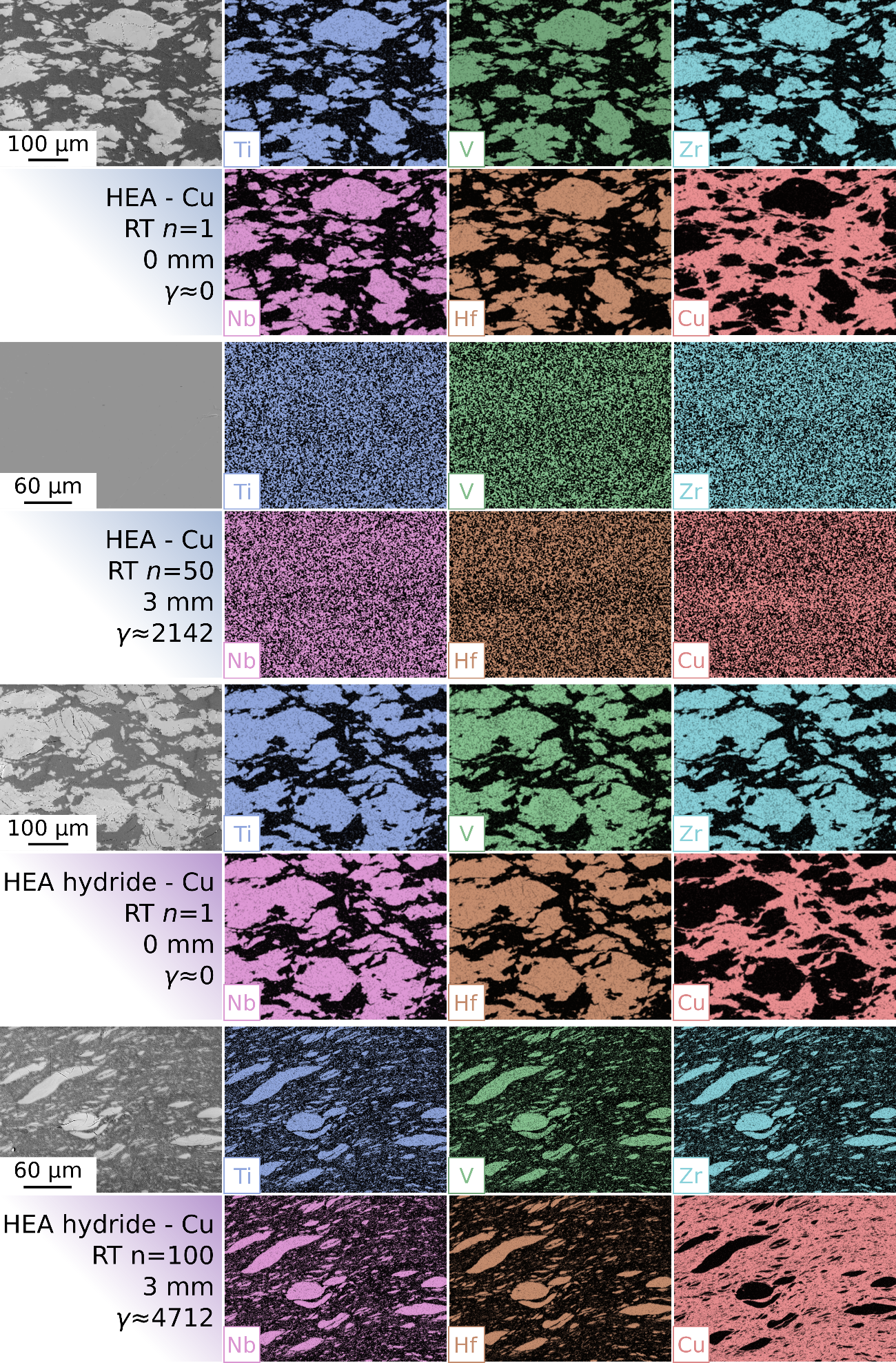
**

**Figure S1:** Secondary electron SEM micrographs and EDX maps of the HEA-Cu and HEA hydride-Cu composites for varying amounts of HPT deformation.

**2. XRD characterization**


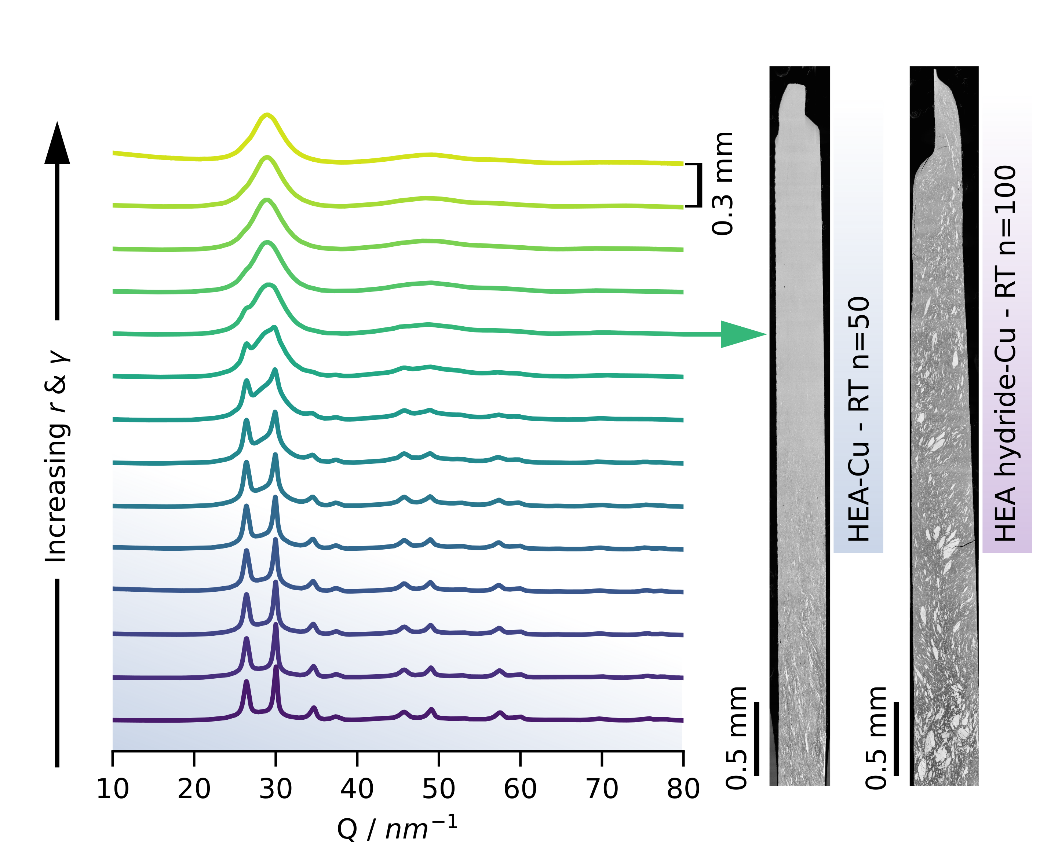


**Figure S2:** Cross-sections of the HEA-Cu and the HEA hydride-Cu disks after HPT deformation at RT and *n*= 50 and 100. Radius-specific Synchrotron XRD patterns are plotted for the HEA-Cu composite at the respective positions.

**3. Calorimetric characterization**

DSC measurements (heating rate of 10 and 20 K min^−1^) were conducted to better understand the metastable character of the materials prepared in this study. **Figure S3** depicts the DSC curves of the HEA-Cu and HEA hydride-Cu composites.

As seen in **Figure S3 (a)** for the HEA-Cu composites, at *n*= 1 and n = 10, two relatively broad peaks are visible at 500 and 600 °C, while at *n*= 50, these become two pronounced and well-defined peaks. These are associated with the decomposition of the solid-solution/amorphous phase. SEM and EDX investigations of the DSC samples, given in **Figure S4,** show decomposition into a complex multi-phase material containing a Cu-Zr-rich phase (Phase 1, large grains in **Table S1**), a Nb-V-rich phase (Phase 2, globular grains in **Table S1**), and a Cu-rich intergranular phase (Phase 3, Intergranular in **Table S1**) surrounded by a Ti-enriched interphase (Phase 4, Interphase in **Table S1**). The exact chemical compositions are given in **Table S1.**

The DSC curves of the less deformed hydride composites at *n*= 1 and 10, given in **Figure S3 (b),** exhibit three convoluted endothermic peaks related to dehydrogenation.^[1]^ The peaks become less pronounced and shift to higher temperatures with increasing strain. This suggests that embedding the HEA hydride in a rigid Cu matrix stabilizes the hydride, which then desorbs hydrogen only at higher temperatures. No distinct endothermic signal was obtained for the most severely deformed sample at *n*= 100. On the contrary, broad exothermic peaks were observed above temperatures of about 500 °C. Although SPD could hinder the hydride decomposition, it is unlikely that it is stable up to 1000 °C. The HPT-deformed nanocrystalline material is in a highly metastable state. Upon heating to high temperatures, interdiffusion and associated phase transformations will occur as soon as the stabilizing effect of the hydrogen/hydride is removed. Such exothermic processes could surpass and mask the endothermic hydrogen desorption. This highlights the stabilizing character of hydrogen for this particular nanostructure. The microstructure after DSC measurements is given in **Figure S5,** showing a similar microstructure to the HEA-Cu composite without hydrogen.


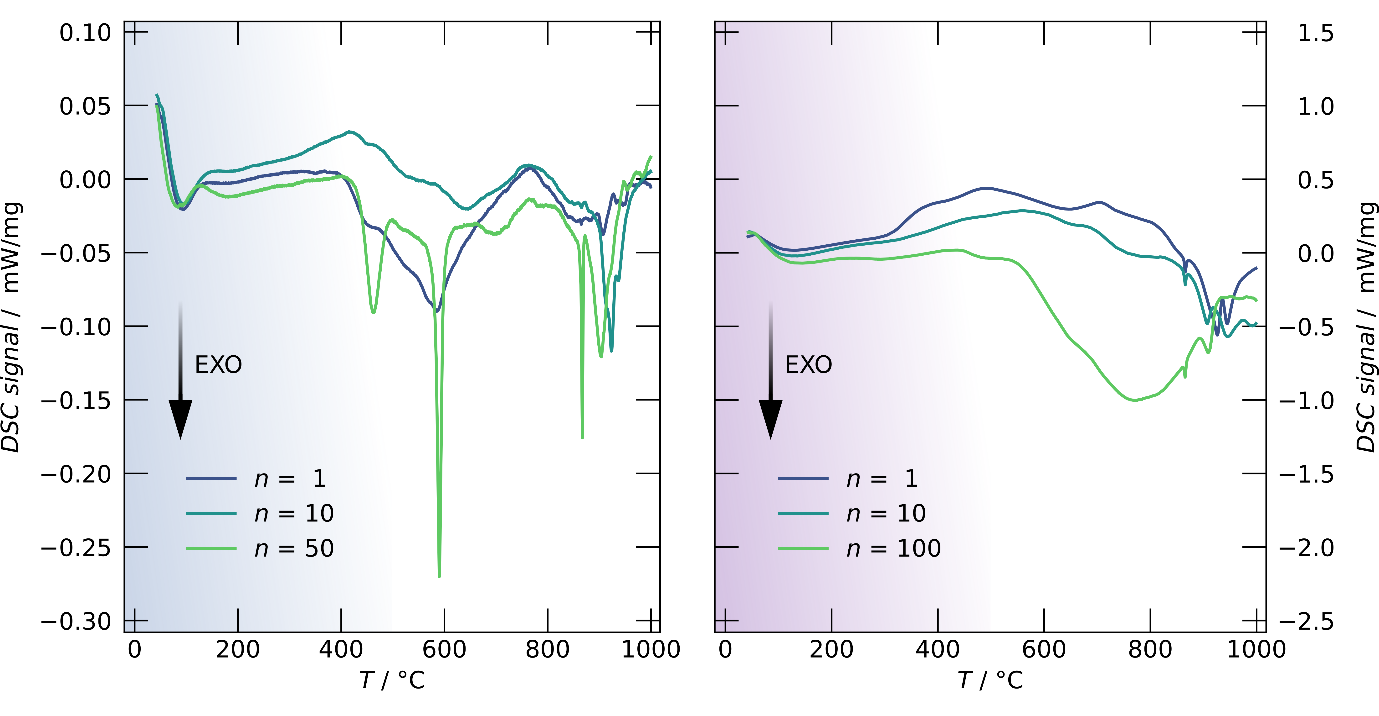


**Figure S3:** DSC measurements of the (a) HEA-Cu composite at *n*= 1, 10, and 50 (10 K min^−1^) and (b) the HEA hydride-Cu composite at *n*= 1, 10, and 100 (20 K min^−1^).

**
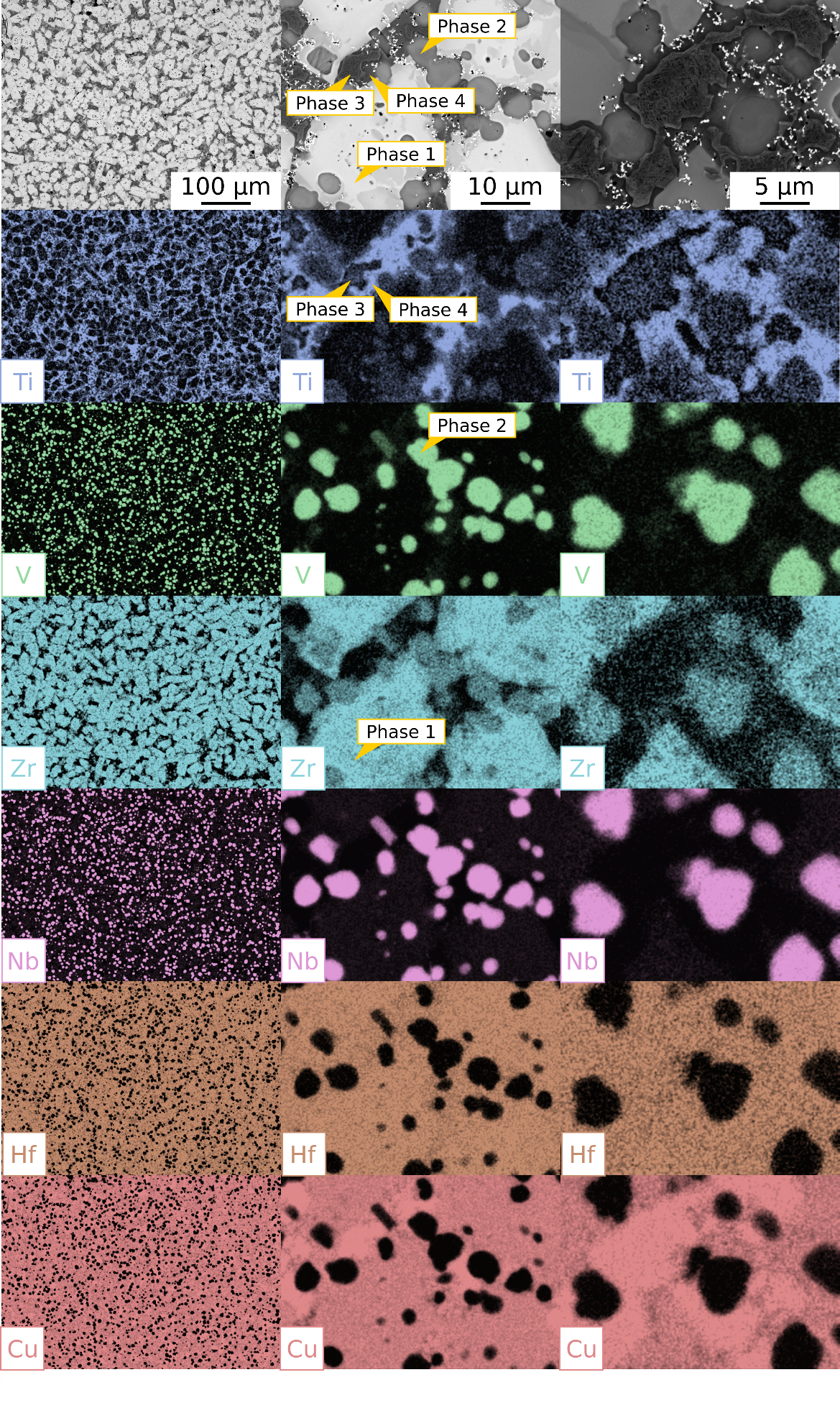
**

**Figure S4:** BSE SEM micrographs and associated EDX maps of the HEA-Cu composite with *n*= 50 after DSC characterization (heated to 1000 °C).

**
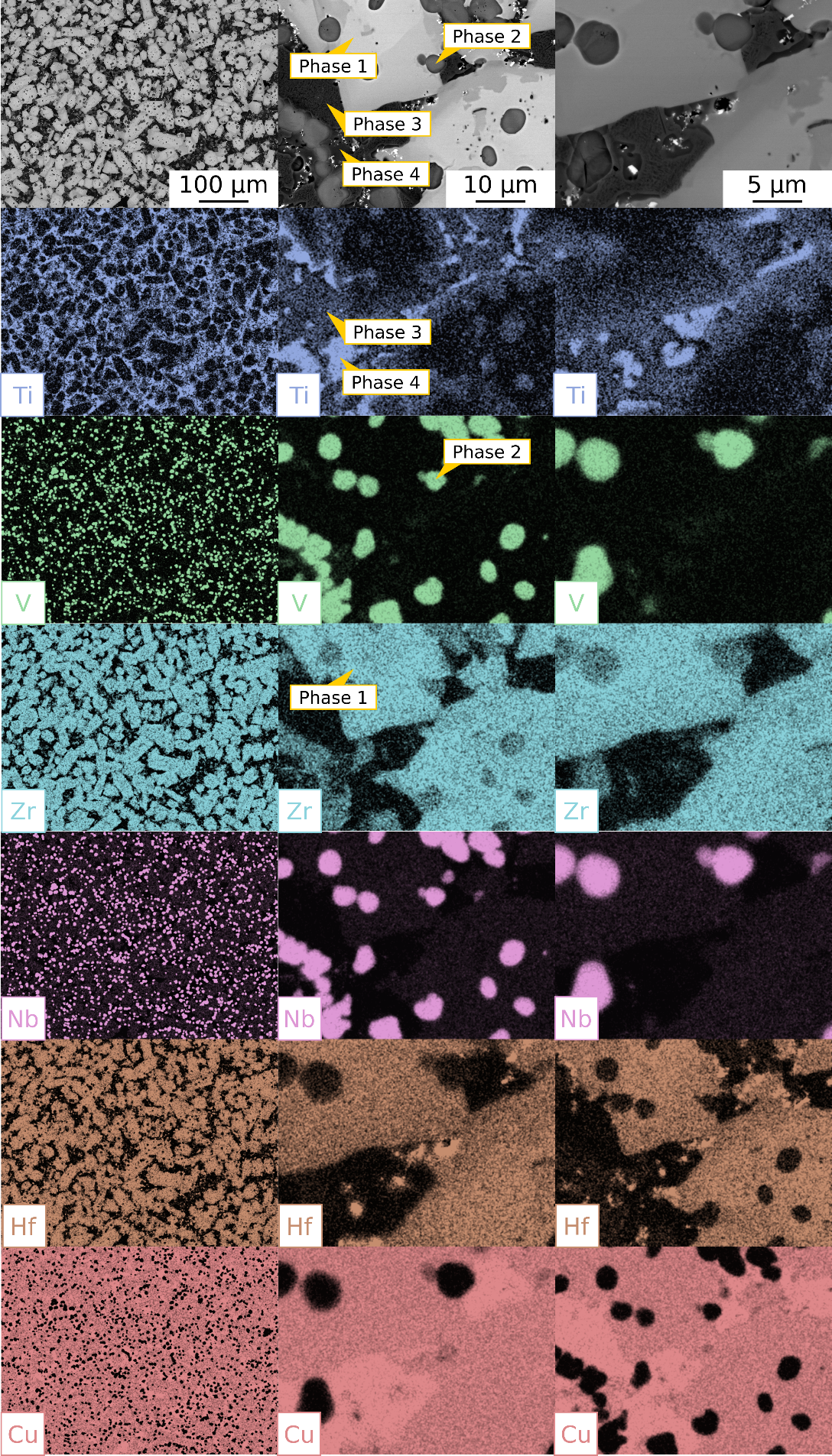
**

**Figure S5:** BSE SEM micrographs and associated EDX maps of the HEA hydride-Cu composite at *n*= 100 after DSC characterization (heated to 1000 °C).

**4. Mechanical characterization**

Hardness measurements of the HEA and Cu reveal distinct strength differences. The HEA registers 6.1 ± 0.8 GPa, while the HEA hydride is slightly stronger at 6.8 ± 0.5 GPa. In contrast, Cu is significantly softer at 1.4 ± 0.1 GPa. These results indicate that the hardness differences between the HEA / HEA hydride and Cu are consistent in the hydrogenated and dehydrogenated states. It should be noted that cracking at the indents was observed, especially for the hydrides, so the reported hardness values are semi-quantitative estimations. Therefore, a strength difference of 5 was assumed for both the HEA and HEA hydride systems in the Monte Carlo simulation; see below for details.

**5. Thermodynamic Considerations**

The thermodynamic considerations are based on the work of Yang and Zhang on predicting the stability of HEAs.^[2]^ Using the enthalpies of mixing of the respective binary liquid alloys, $\Delta H_{\mathrm{AB}}^{mix}$, given in **Table S3**, the regular solution interaction parameter $\Omega_{\mathrm{ij}}$ was calculated using

$\Omega_{\mathrm{ij}}=4 \Delta H_{\mathrm{AB}}^{mix}$. (S1)

Based on this, the enthalpy of mixing of a multi-component alloy can be estimated from

$\Delta H_{\mathrm{mix}}= \sum_{i=1,i\neq j}^{n} \Omega_{\mathrm{ij}} c_{i} c_{j},$ (S2)

with $c_{i}$ and $c_{j}$ being the atomic percent of the alloy system. The entropy of mixing $\Delta S_{\mathrm{mix}}$ was estimated based on

$\Delta S_{\mathrm{mix}}= -R\sum_{i=1}^{n} ( c_{i}\ln c_{i}),$ (S3)

with *R* being the ideal gas constant, i.e., 8.314 J K^−1^ mol^−1^. From the enthalpies and entropies, the Gibbs free energy associated with alloy formation and, as an extension, mechanical alloying can be evaluated using

$\Delta G_{\mathrm{mix}}=\Delta H_{\mathrm{mix}}-T \Delta S_{\mathrm{mix}}$ (S4)

with *T* being the deformation temperature during HPT, i.e., 300 K. The values for Δ*H*_mix_, Δ*S*_mix_, and Δ*G*_mix_ calculated using this methodology are provided in **Table S4** for TiVZrNbHf and TiVZrNbHf-Cu,^[2,3]^ together with the respective hydrogen interactions, i.e., Cu-H and TiVZrNbHf-H.^[4–6]^

**Table S3:** $\Delta H_{\mathrm{AB}}^{mix}$ of all elemental pairs in kJ mol^−1^ taken from Takeuchi and Inoue.^[3]^

|  | Ti | V | Zr | Nb | Hf | Cu |
| --- | --- | --- | --- | --- | --- | --- |
| Ti | - | -2 | 0 | 2 | 0 | -9 |
| V | - | - | -4 | -1 | -2 | 5 |
| Zr | - | - | - | 4 | 0 | -23 |
| Nb | - | - | - | - | 4 | 3 |
| Hf | - | - | - | - | - | -17 |
| Cu | - | - | - | - | - | - |

**Table S4:** Δ*H*_mix_, Δ*S*_mix_, and Δ*G*_mix_ of the various metal-metal and metal-hydrogen interactions in the TiVZrNbHf-Cu-H systems computed using the enthalpy values in **Table S3** and **Equations S1-S4**.^[2–6]^ The Δ*H*_mix_ values per atom were used for the Monte Carlo simulations.

| Type of interaction | ΔH_mix_ / kJ mol^−1^(H) | ΔS_mix_ / J K^−1^ mol^-1^ (H) | ΔG_mix_ (300 K)  / kJ mol^−1^ | ΔH_mix_ / kJ atom^−1^ |
| --- | --- | --- | --- | --- |
| Hea-Cu (A-B) | −7.70 | 10.61 | −10.89 | −1.28·10^−23^ |
| Hea (A-A) | 0.16 | 13.38 | −3.85 | 2.66·10^−25^ |
| Cu (B-B) | 0.00 | 0.00 | 0.00 | 0.00 |
| Hea-H (A-H) | −29.50 | −41.00 | −17.20 | −4.90·10^−23^ |
| Cu-H (B-H) | 42.45 | −50.48 | 57.60 | 7.05·10^−23^ |

**6. Monte Carlo Simulations**

A model based on Monte Carlo simulations was developed to validate the impact of hydrogen and varying hydrogen affinities on the microstructural evolution during HPT. Simulations were conducted under conditions with and without hydrogen to compare the respective effects.

A (50x50) lattice representing the atom positions and occupied with A atoms (HEA - high hydrogen affinity) and B atoms (Cu - low hydrogen affinity) was defined in an initial step. Interactions were limited to nearest neighbors, i.e., each atom interacted with the four surrounding lattice sites. Based on the mixing enthalpies Δ*H*_mix_, calculated using **Equation S2** and given in **Table S4**, the A-A and A-B interaction energies were estimated with 2.66·10^−25^ kJ per atom and −1.28·10^−23^ kJ per atom, respectively. Before being used as MC simulation input parameters, these values have to be normalized by the number of bonds, i.e., divided by a factor of 4. B-B interactions (Cu-Cu) were set to 0.

Each Monte Carlo (MC) step included a defined probability for diffusion and shear, respectively. The diffusion step swaps two randomly chosen neighboring atoms. Shear results in the movement by one lattice position along a randomly selected horizontal or vertical line in the grid to mimic the HPT deformation. Periodic boundary conditions were imposed on both diffusion and shear steps.

The probability of shear or diffusion attempts was calculated based on the strain rate and diffusion coefficient during HPT. Notably, the diffusion coefficient can change significantly in SPD-deformed materials compared to the bulk coarse-grained material, and could therefore only be estimated in this study. Therefore, it was varied in a wide range from 10^−30^ to 10^−10^m^2^ s^−1^ to get a complete picture, with realistic values most likely in the range of 10^−18^-10^−21^m^2^ s^−1^.^[7–11]^ Based on the diffusion coefficient, the jump frequency $\Gamma$ can be calculated using

$\Gamma=\frac{4 D}{\lambda^{2}}$ (S5)

With $\lambda$ being the jump distance (estimated as 2.9 Å), *D* the diffusion coefficient, and the factor of 4 accounting for the 2D grid geometry.^[12]^ This frequency should not be confused with the attempt frequency ν (≈10^13^) often referred to in diffusion, as an MC attempt already represents a successful jump surmounting the initial activation barrier.

At 1.27 rpm, a radius of 4 mm, and a disk thickness of 0.5 mm, a shear strain *γ* of 1.06 is achieved within 1 s. In the MC simulations with a grid of 50x50, a shear strain of 1 is equivalent to 50 shear steps. Consequently, 53.2 shear steps per second result in this shear strain rate. The resulting jump frequencies $\Gamma$ and shear rates yield the respective (relative) probabilities of diffusion and shear attempts in the system. Two types of simulations were performed, allowing only horizontal shear or giving a 20 % chance of a vertical shear event, respectively. The latter mimics less ideal shear conditions or turbulent plastic flow during HPT.^[13]^

Additionally, each diffusion attempt was accepted or declined based on the Metropolis criterion,^[14]^ with the temperature set to 300 K and respective energy changes calculated using the Δ*H*_mix_ in **Table S4**.

Each shear attempt was accepted with a probability linearly scaled by the types of atoms present along the shear line. This simulated the flow strength differences between Cu and the HEA and allowed us to model deformation localization and its interaction with mechanical alloying/diffusion. However, unlike diffusion, each shear attempt is repeated until a shear event occurs, i.e., a particular shear strain rate is enforced. Both HEA and HEA hydride were assumed to have five times the flow strength of the Cu phase, which is in line with experimental observations based on microhardness measurements mentioned above.

The total energy was calculated by summing up all nearest-neighbor interactions. The ordering/mixing was assessed by examining the types of neighbors for each atom. A *mixing index* *i*_mix_ was defined as

$i_{mix}=\frac{1}{4M^{2}}\sum_{i=1}^{M} \sum_{j=1}^{M} \sum_{k \in N(i,j)} (1-\delta\left( G\left( i,j \right), G\left( k \right) \right))$ (S6)

With *M* being the grid size along the two dimensions, *G*(*i,j*) the atom at position (*i,j*), *N*(*i,j*) represents the four neighbors of every site, accounting for periodic boundary conditions. *δ* is the Kronecker delta. A value of 0 indicates no A-B pairs, 0.5 is an even distribution with 50% A-B and 50% A-A/B-B pairs, and 1 exclusively A-B pairs. Values 0 and 1 correspond to highly ordered states, though distinctly different, while 0.5 signified a random atom distribution.

Hydrogen was included by defining a second, superimposed sub-lattice. Hydrogen was positioned at the position of the A atoms to reflect the hydride. The A-H and B-H interactions were estimated based on the enthalpy of the hydride formation of TiVZrNbHf and the enthalpy of hydrogen dissolution in Cu. Using the values in **Table S4**,^[4–6]^ the former amounts to −4.90·10^−23^ kJ atom^−1^, and the latter to 7.05·10^−23^kJ atom^−1^.

The hydrogen diffusivity was estimated to be significantly faster than the metal diffusion^[15]^ and was set to 10 times the latter's value. An even higher ratio was avoided to reduce the computational demand of the MC simulation.

The in-depth results of the MC simulations for the HEA-Cu and HEA hydride-Cu systems are presented in **Figures S6** to **S13**.

**Figures S6-7** show the HEA-Cu results for horizontal shear only, while **Figures S8-9** show the results for horizontal and vertical shear. **Figure S6 (a)** shows representative grids for different diffusion coefficients *D*, while the evolution of calculated energies and mixing indices is illustrated as a function of **(b,c)** MC steps and **(d,e)** shear strain. The simulations demonstrate convergence, with consistent results across multiple MC runs. Large diffusion coefficients result in significant mixing and an ordered structure, reflected by a mixing index near 1. Conversely, at lower diffusion coefficients, where shear probability increases, the mixing index approaches 0.5, indicating a random solid solution. **Figure S7** reveals a uniform distribution of shear events across the grid, showing that interdiffusion mitigates the initial tendency for shear localization and promotes a more homogeneous strain distribution. The same trends are visible in **Figures S8-9**, although these results highlight the more efficient mixing and more evenly distributed plastic deformation induced by the multi-directional shear.


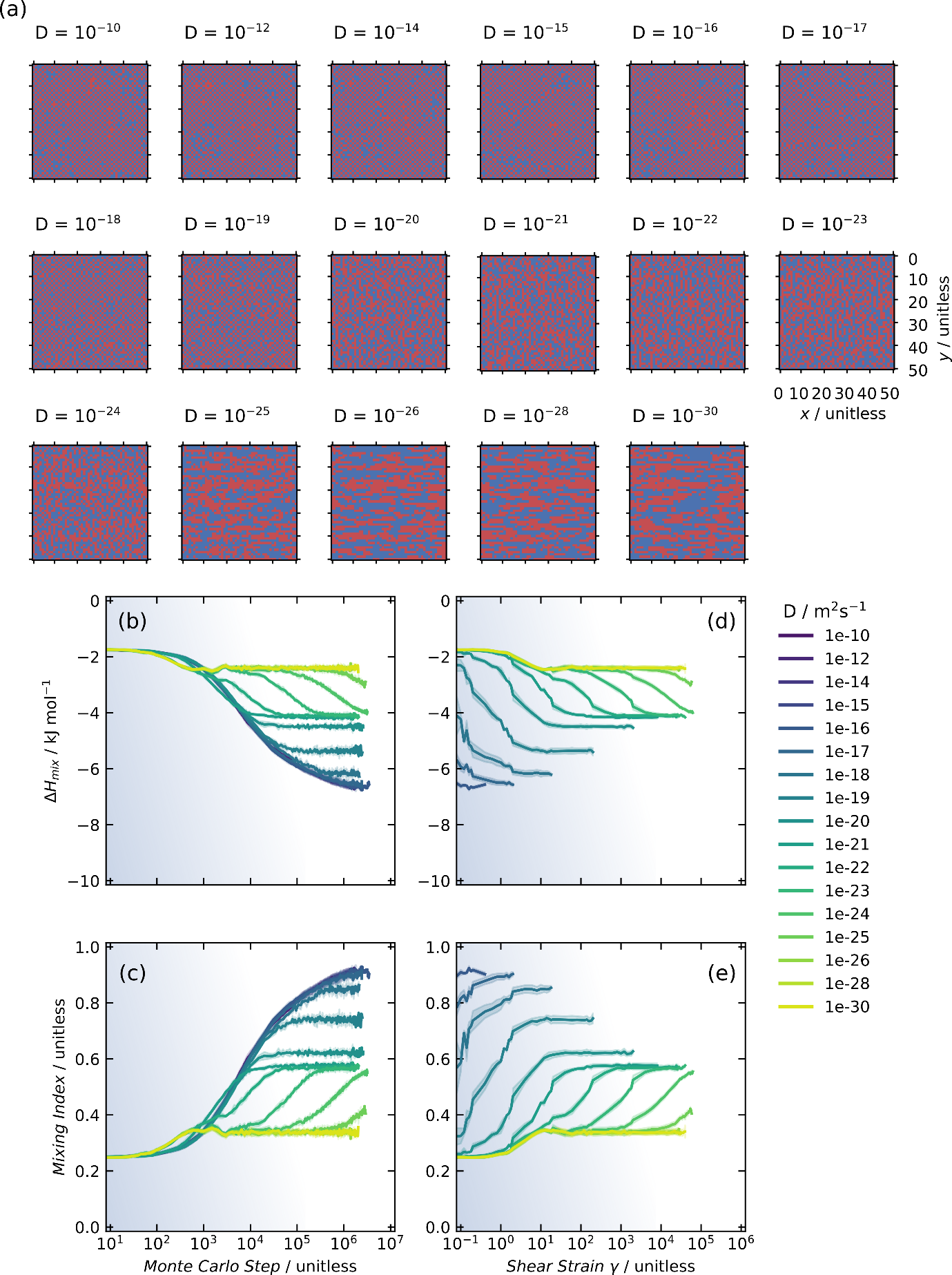


**Figure S6:** Results of the Monte Carlo simulations of the HEA-Cu composite without any hydrogen and assuming different diffusion coefficients (in m^2^ s^−1^). (a) Final grids and calculated energies and mixing indices as functions of (b,c) MC steps and (d,e) shear strain. Only horizontal shear events were allowed.


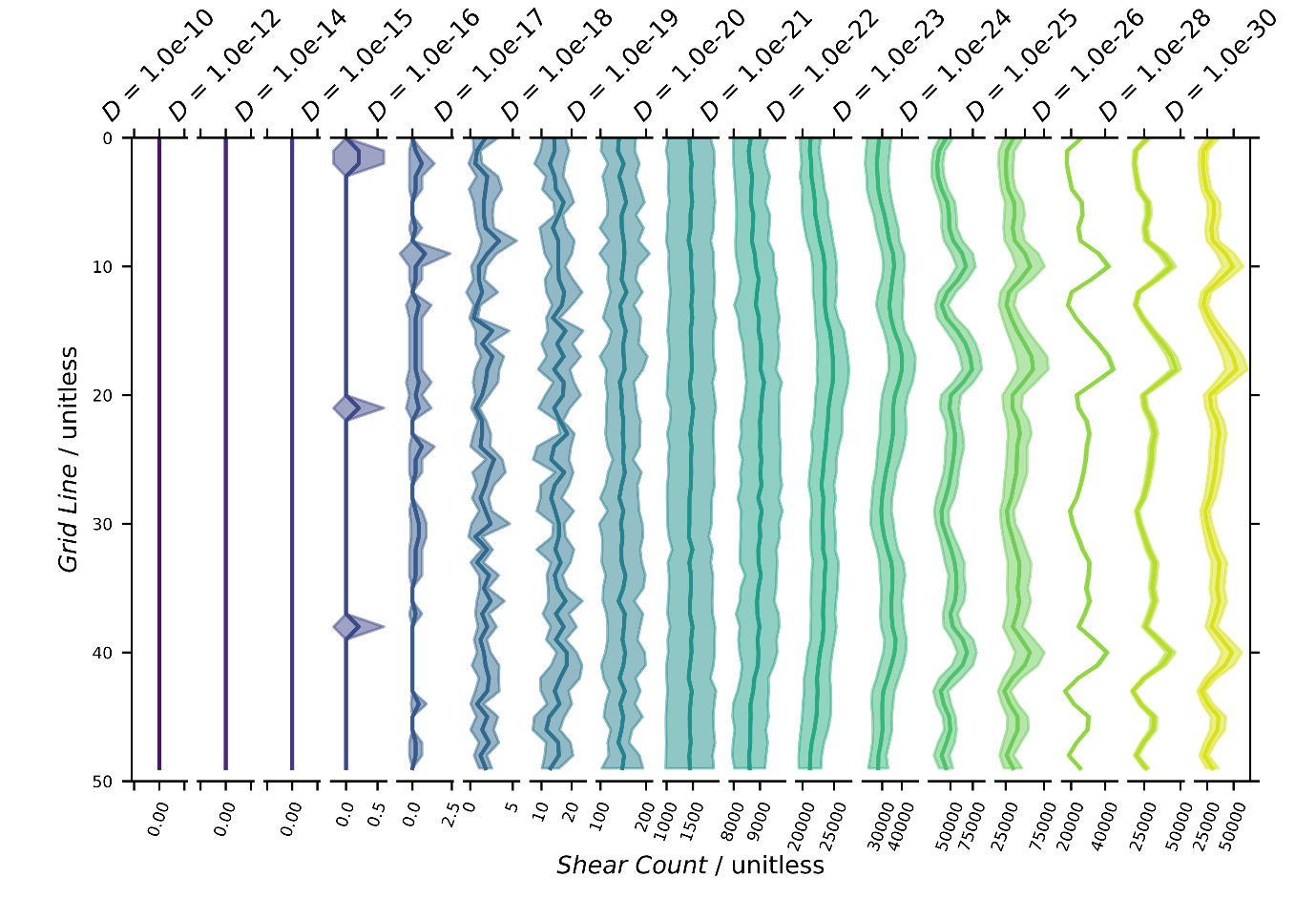


**Figure S7:** Distribution of the shear steps (horizontal only) in HEA-Cu composites derived from the Monte Carlo simulations assuming different diffusion coefficients (in m^2^ s^−1^). Only horizontal shear events were allowed.

**
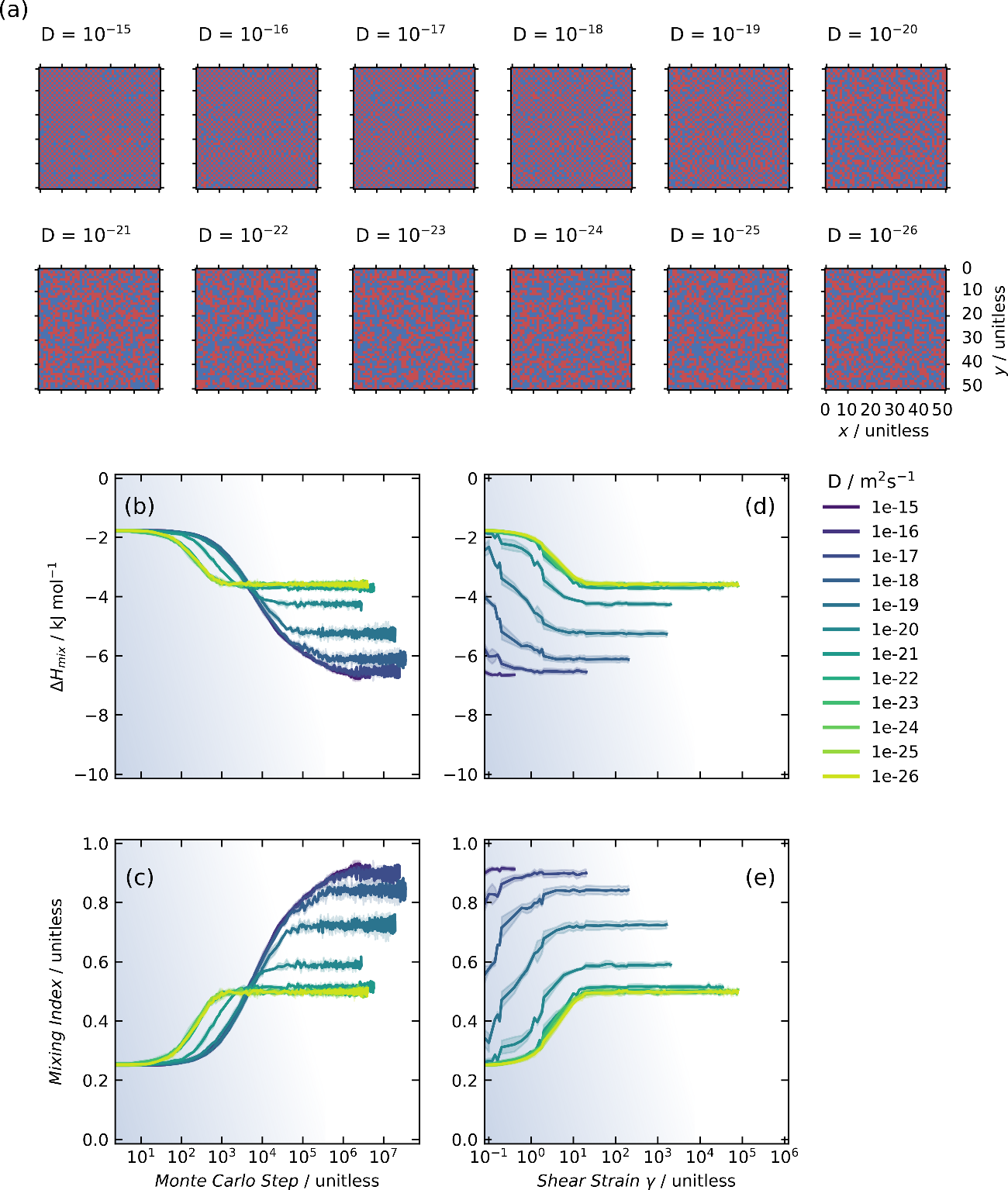
Figure S8:** Results of the Monte Carlo simulations of the HEA-Cu composite without any hydrogen and assuming different diffusion coefficients (in m^2^ s^−1^). (a) Final grids and calculated energies and mixing indices as functions of (b,c) MC steps and (d,e) shear strain. In addition to the horizontal shear events, there was a 20 % chance of a vertical shear event.


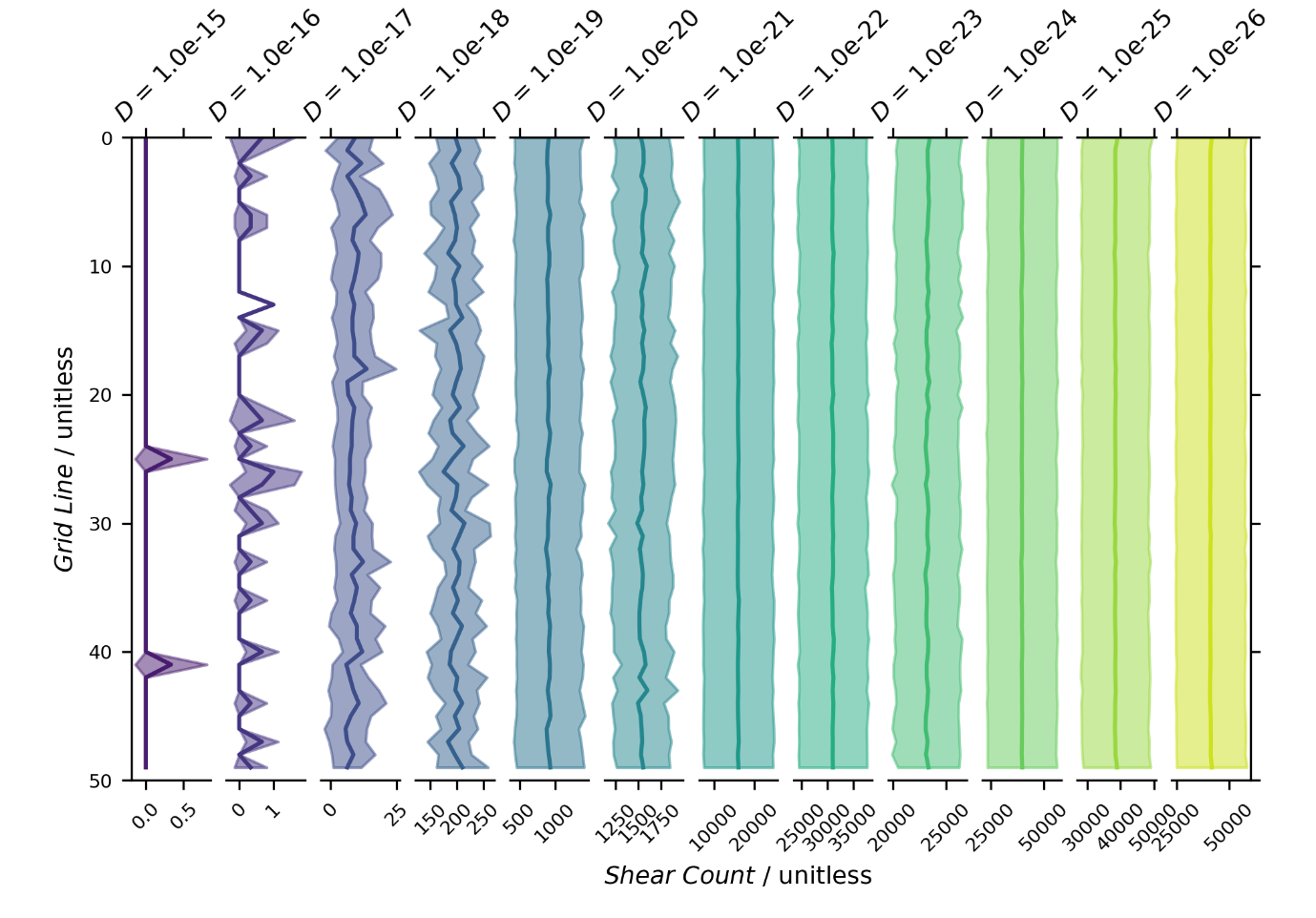


**Figure S9:** Distribution of the shear steps (horizontal only) in HEA-Cu composites derived from the Monte Carlo simulations assuming different diffusion coefficients (in m^2^ s^−1^). In addition to the horizontal shear events, there was a 20 % chance of a vertical shear event.

**Figures S10-S11** show the HEA hydride-Cu results for horizontal shear only, while **Figures S12-S13** show the results for horizontal and vertical shear. **Figure S10 (a)** presents metal and hydrogen sub-grids at varying diffusivities for the HEA hydride-Cu system. At high diffusivities (low shear probability), phase separation persists with a coarse structure. As shear probability increases (lower *D*), the microstructure becomes lamellar, reflecting a strong tendency for phase separation even under high shear rates. **Figure S10** shows again the evolution of calculated energies and mixing indices as a function of **(b,c)** MC steps and **(d,e)** shear strain. **Figure S11** highlights pronounced strain localization in this regime. Mechanical intermixing dominates at very low diffusivities and high shear probabilities, leading to a higher mixing index. As seen in **Figure S11**, strain localization is significantly reduced in this range. Again, the same trends are visible in **Figures 12-13**, although these results highlight the more efficient mixing and more evenly distributed plastic deformation induced by the multi-directional shear.

**
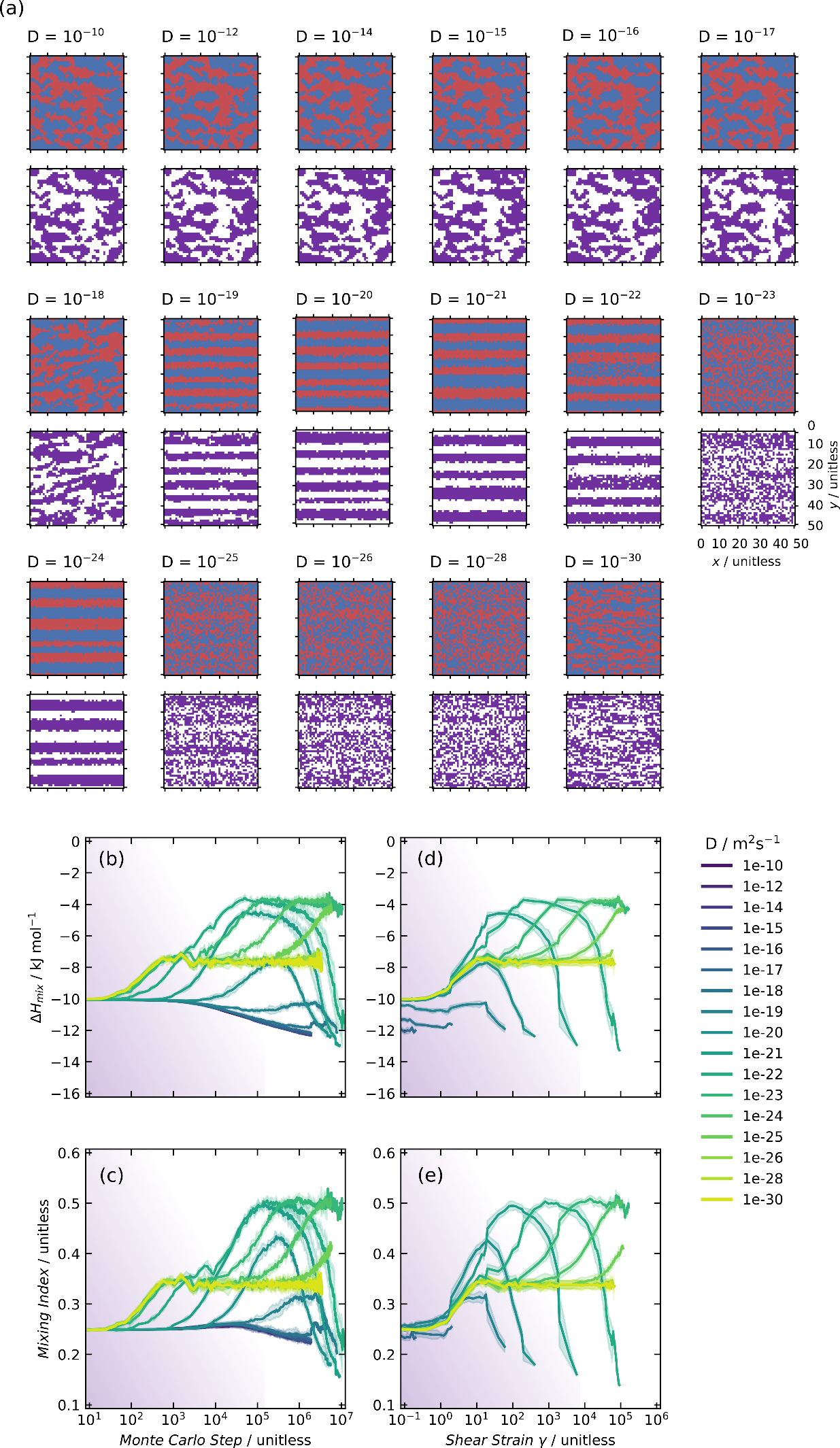
**

**Figure S10**

**
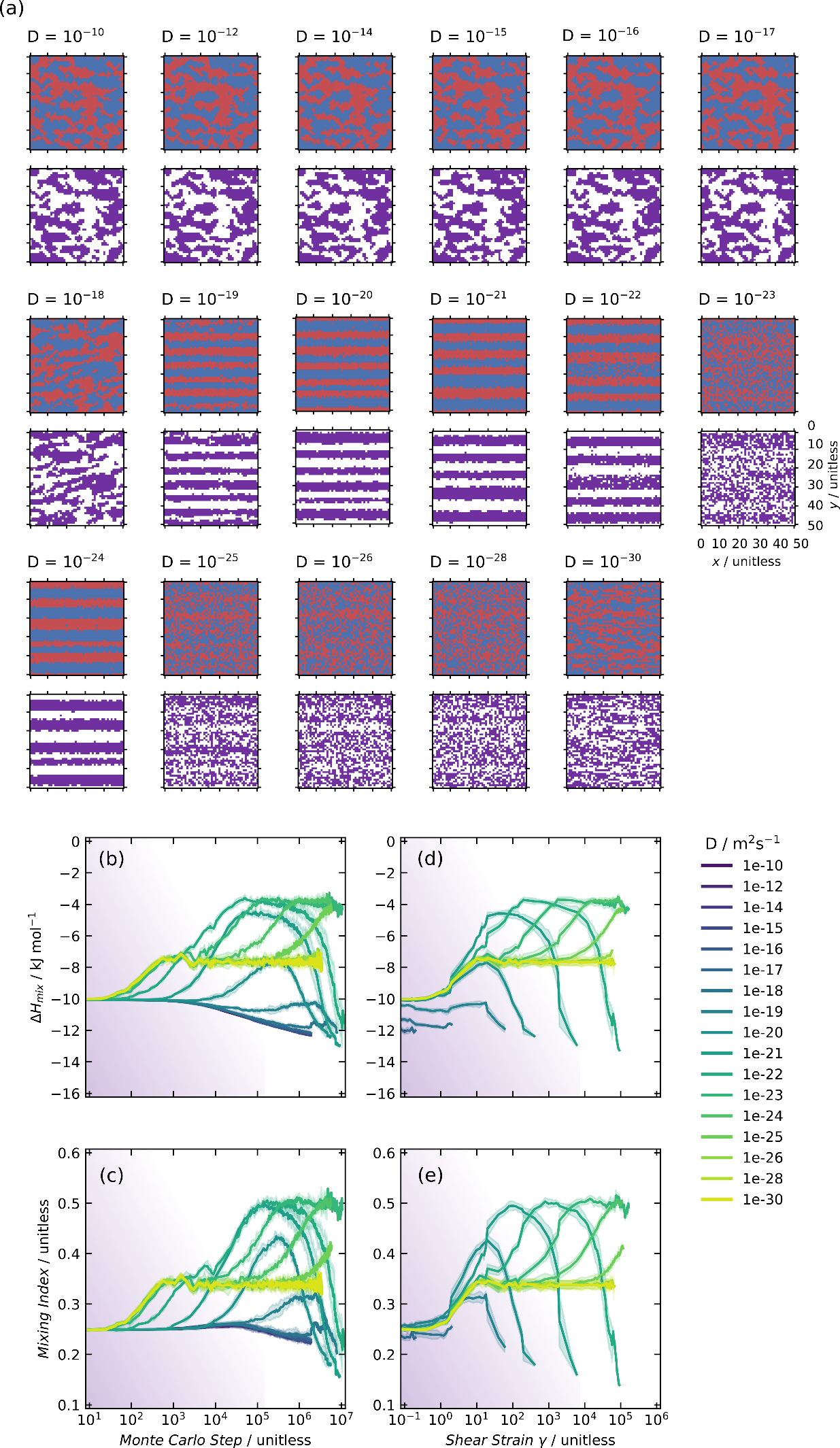
Figure S10:** Results of the Monte Carlo simulations of the HEA hydride-Cu composite, i.e., with hydrogen, and assuming different diffusion coefficients (in m^2^ s^−1^). (a) Final grids and calculated energies and mixing indices as functions of (b,c) MC steps and (d,e) shear strain. Only horizontal shear events were allowed. (continued)


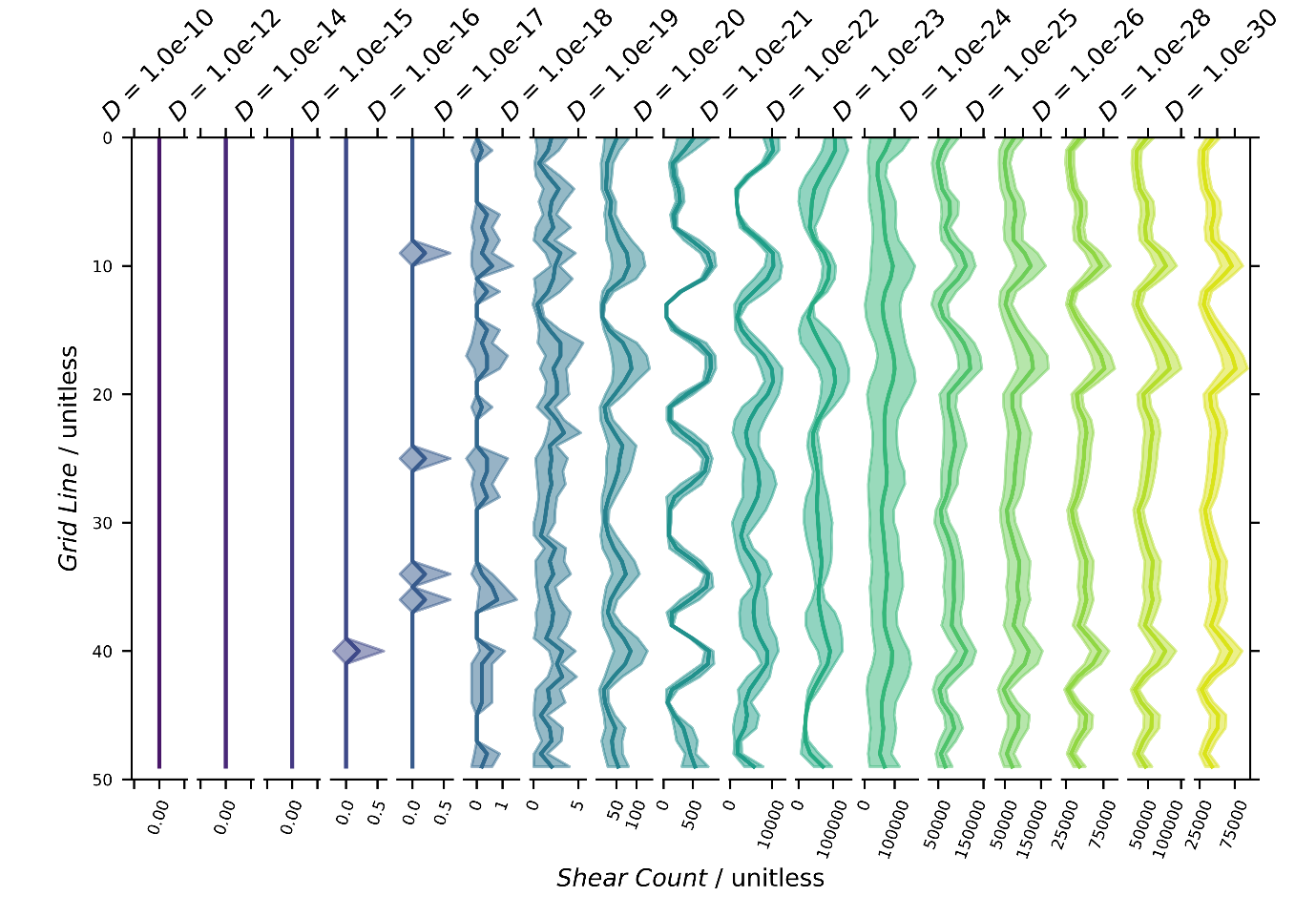


**Figure S11:** Distribution of the shear steps (horizontal only) in HEA hydride-Cu composites derived from the Monte Carlo simulations assuming different diffusion coefficients (in m^2^ s^−1^). Only horizontal shear events were allowed.

**
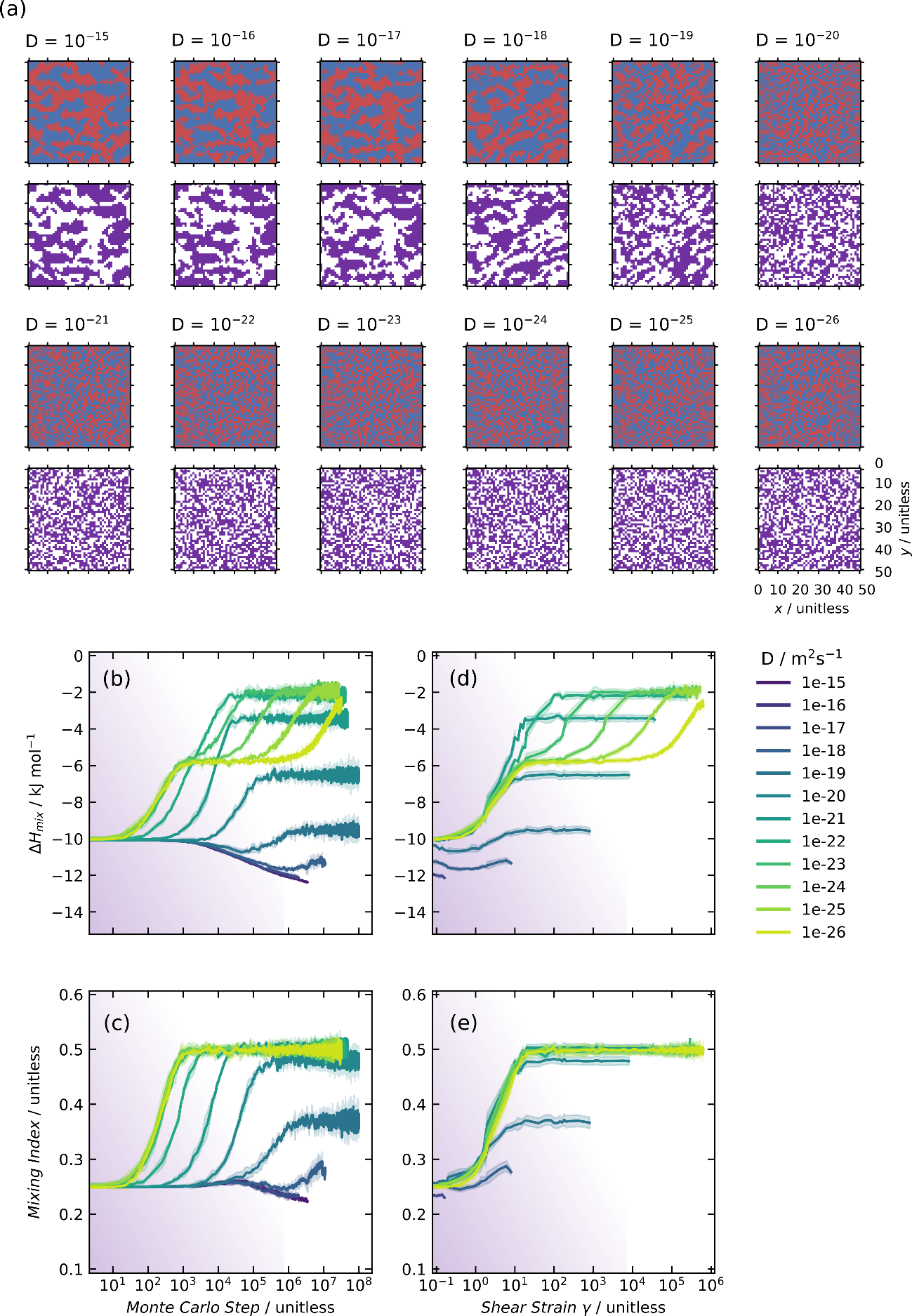
Figure S12:** Results of the Monte Carlo simulations of the HEA hydride-Cu composite, i.e., with hydrogen, and assuming different diffusion coefficients (in m^2^ s^−1^). (a) Final grids and calculated energies and mixing indices as functions of (b,c) MC steps and (d,e) shear strain. In addition to the horizontal shear events, there was a 20 % chance of a vertical shear event.

**
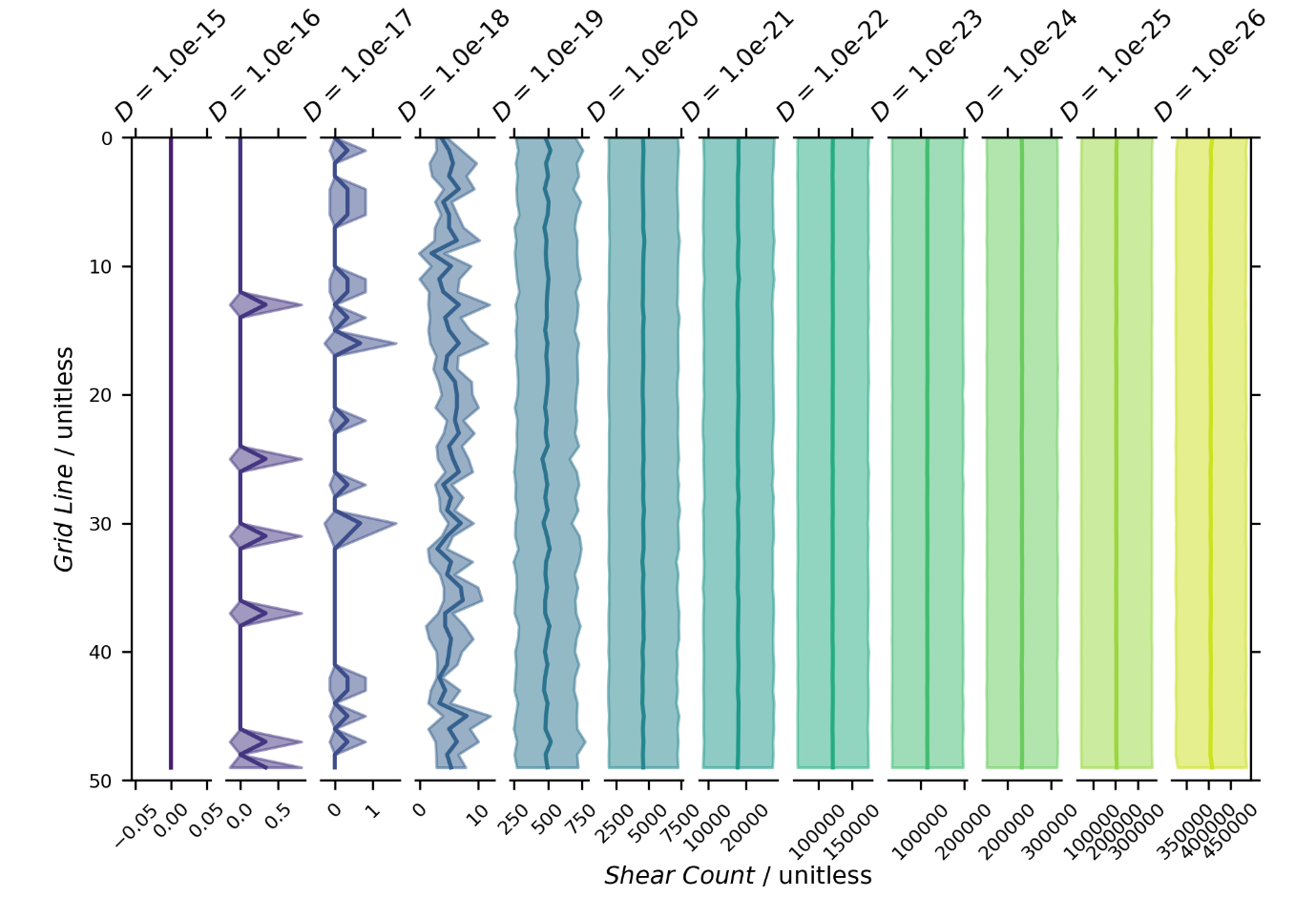
**

**Figure S13:** Distribution of the shear steps (horizontal only) in HEA hydride-Cu composites derived from the Monte Carlo simulations assuming different diffusion coefficients (in m^2^ s^−1^). In addition to the horizontal shear events, there was a 20 % chance of a vertical shear event.

**6. Morris sensitivity analysis of the Monte Carlo simulation**

A Morris sensitivity analysis, implemented using the Python package SALib,^[16,17]^ was performed for the MC simulation. The input parameters are varied in a controlled way, and based on the output, i.e., the mixing index, the mean absolute elementary effects (μ*: a measure of how changes in the input influence the output) and their respective standard deviations (σ: a measure of non-linearity or interactions with other inputs) were determined. Large values imply strong effects on the simulation outcome. The results are given in **Tables S5** and **S6.**

**Table S5:** Results of the Morris sensitivity analysis performed on the MC simulation of the HEA-Cu system. The mixing index was used as the output parameter of the analysis.

| Input parameter | Variation | µ* | σ |
| --- | --- | --- | --- |
| Diffusion coefficient / m^2^ s^−1^ | 10^−10^ - 10^−30^ | 0.514 | 0.217 |
| Shear steps / s^−1^ | 25 - 100 | 0.010 | 0.014 |
| Temperature / K | 300 - 500 | 0.042 | 0.052 |
| A-A Interaction Energy / kJ mol^−1^ | 50 - 200 % | 0.014 | 0.023 |
| A-B Interaction Energy / kJ mol^−1^ | 50 - 200 % | 0.111 | 0.167 |
| Strength B / unitless (Strength A ≡ 1) | 10 - 100 % | 0.012 | 0.018 |

**Table S6:** Results of the Morris sensitivity analysis performed on the MC simulation of the HEA hydride-Cu system. The mixing index was used as the output parameter of the analysis.

| Input parameter | Variation | µ* | σ |
| --- | --- | --- | --- |
| Diffusion coefficient / m^2^ s^−1^ | 10^−10^ - 10^−30^ | 0.208 | 0.193 |
| Shear steps / s^−1^ | 25 - 100 | 0.021 | 0.052 |
| Temperature / K | 300 - 500 | 0.017 | 0.026 |
| A-A Interaction Energy / kJ mol^−1^ | 50 - 200 % | 0.010 | 0.016 |
| A-B Interaction Energy / kJ mol^−1^ | 50 - 200 % | 0.10 | 0.129 |
| A-H Interaction Energy / kJ mol^−1^ | 50 - 200 % | 0.080 | 0.147 |
| B-H Interaction Energy / kJ mol^−1^ | 50 - 200 % | 0.108 | 0.153 |
| Strength B / unitless (Strength A ≡ 1) | 10 - 100 % | 0.009 | 0.013 |

**7. Reported diffusion coefficients**

**Table S7**: Results of various studies in literature about SPD processed metals and metal composites.

| System | Method | Analysis methods | Type | D / m2s-1 | Extrapolated D (300 K) | Reference |
| --- | --- | --- | --- | --- | --- | --- |
| Al-Cu | HPT | Diffusion distances and times ^a)^ | Bulk & gb. diffusion | 2·10^−18^ / 6·10^−17^ (340 K) | - | ^[7]^ |
| Al-Ni | HPT | Diffusion distances and times ^a)^ | Bulk & gb. diffusion | 3·10^−19^ (300 K) | - | ^[8]^ |
| Co-Cu | HPT | Diffusion distances and times ^a)^ | Bulk & gb. diffusion | 5·10^−19^ (300 K) | - | ^[18]^ |
| Al-Ni | HPT | Diffusion distances and times ^a)^ | Bulk & gb. diffusion | 2·10^−14^ (573 K) | - | ^[19]^ |
| CoCrFeNiMn | ECAP | ^57^Co tracer diffusion | Gb. diff. | 5·10^−18^ (373 K) | 2·10^−19^ | ^[9]^ |
| Cu-Zr | ECAP | ^63^Ni tracer diffusion | Gb. diff. | 7·10^−18^ / 7·10^−15^ (424 K) | 3·10^−20^ | ^[11]^ |
| Ni | ECAP | ^63^Ni Tracer diffusion | Gb. diff. | 3·10^−19^ (331 K) | 5·10^−20^ | ^[10]^ |
| Mo | HPT | Ni diffusion profile (Auger) | Gb. diff. | 2·10^−16^ / 10^−13^ (973 K) | 3·10^−19^ | ^[20]^ |
| Ni (Cu) | ECAP / Electrodep. | Cu Profiles | Gb. diff. | 3·10^−17^ / 5·10^−15^ (398 K) | 7·10^−17^ | ^[21]^ |
| Cu | HPT | Ag Radiotracer | Open porosity | 7·10^−15^ (≈ RT ) | - | ^[22]^ |
| Ni | HPT | Co Radiotracer | Gb. diff. | 8·10^−18^ (393 K) | 6·10^−23^ | ^[23]^ |
| CoCrFeNiMn | AM | ^63^Ni Radiotracer | Gb. diff. | 2·10^−18^  - 7·10^−18^ | - | ^[24]^ |
| Nb-Ti | HPT | Diffusion distances and times ^a)^ | Bulk & gb. diffusion | 3·10^−20^ | - | ^[25]^ |
| Mg-Al | HPT | Diffusion distances and times ^a)^ | Bulk & gb. diffusion | 10^−14^  - 3·10^−19^ | - | ^[26]^ |

^a)^ Estimation of the diffusion coefficients based on the observed diffusion distances (i.e., grain size) and estimated diffusion times (i.e., deformation times).

**8. Estimation of the (coarse-grained) diffusion coefficients**


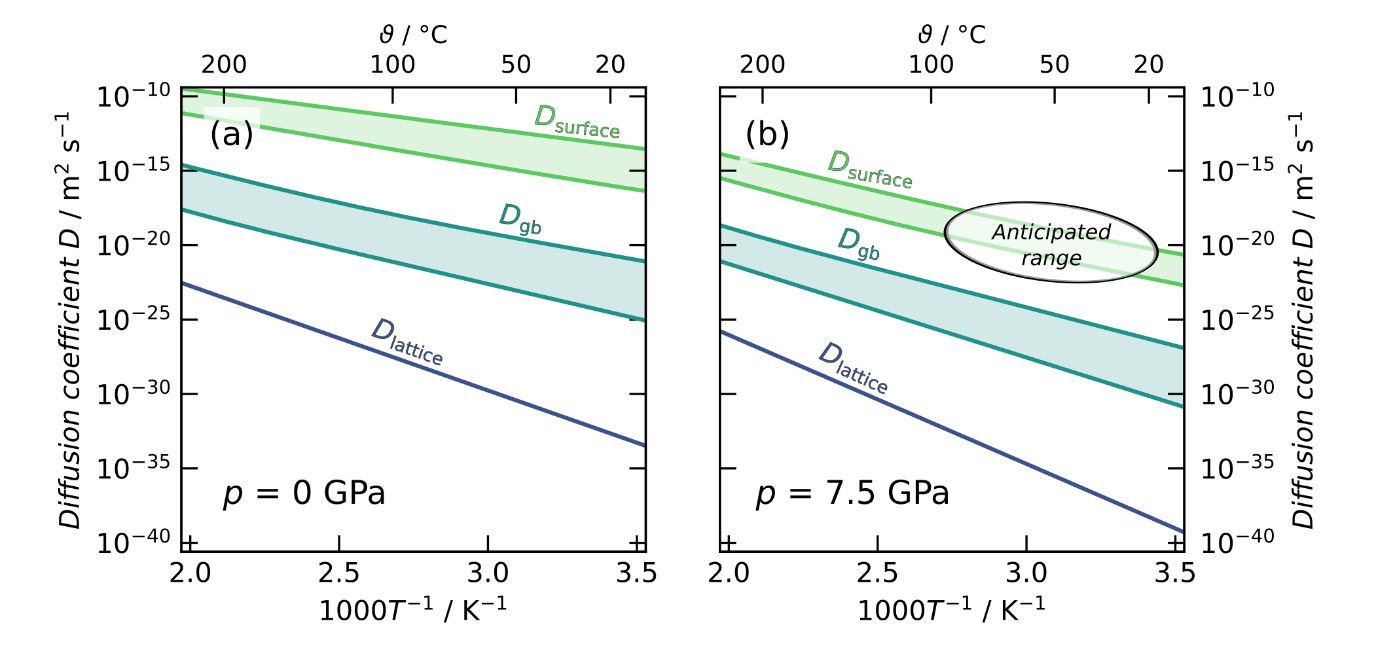
**Figure S14:** Lattice, grain boundary, and surface diffusion coefficients calculated for the TiVZrNbHf-Cu system under 0 GPa and 7.5 GPa pressure. Grain boundary and surface diffusion can be calculated with the assumptions proposed by Oh-ishi et al. (0.33·Q_lattice_ ≥ Q_surface_ ≥ 0.25·Q_lattice_; 0.66·Q_lattice_ ≥ Q_gb_ ≥ 0.50·Q_lattice_).^[7]^

An estimation of the diffusion coefficient of the TiVZrNbHf and Cu system was conducted using ^[12,27]^

$D=D_{0}\exp\left( -\frac{Q-p V_{f}}{RT} \right)$ (S7)

with *D*_0_ being the preexponential factor, *Q* the activation energy of the diffusional process, *p* the pressure applied during HPT, and *V*_f_ the activation volume for diffusion. The values used for the calculations are given in **Table S8**, together with the respective references.

**Table S8:** Material parameters used for calculating the diffusion coefficients plotted in Figure S14.

|  | *D*_0_ / m^2^s^−1^ | *Q*/ kJ mol^−1^ | *p* / GPa | *V*_f_ / m^3 b)^ |
| --- | --- | --- | --- | --- |
| TiVZrNbHf | 4.77·10^−9 a)[28]^ | 134.2 ^[28]^ | 7.5 (exp. param) | 4.2·10^−6 c)^ ^[29]^ |
| Cu | 7.80·10^−5 [30]^ | 200.7 ^[30]^ | 7.5 (exp. param) | 5.7·10^−6 [29]^ |

^a)^ The diffusion coefficient of Zr in TiVZrNbHf was used.
^b)^ The atomic activation volume was calculated using the atomic volumes and respective correction factors according to the given references.
^c)^ The correction factor for bcc V was used to calculate the HEA activation volume.

From the HEA and Cu values, the composite TiVZrNbHf-Cu system diffusion coefficients were estimated based on a Darken relationship ^[12,27]^

$D_{HEA-Cu}={n_{HEA} D}_{HEA}+ n_{Cu} D_{Cu}$ (S8)

*D* are the respective diffusion coefficients, and *n* the respective atomic ratios (38 : 62 = HEA : Cu).

9. Powder and starting material characterization

**
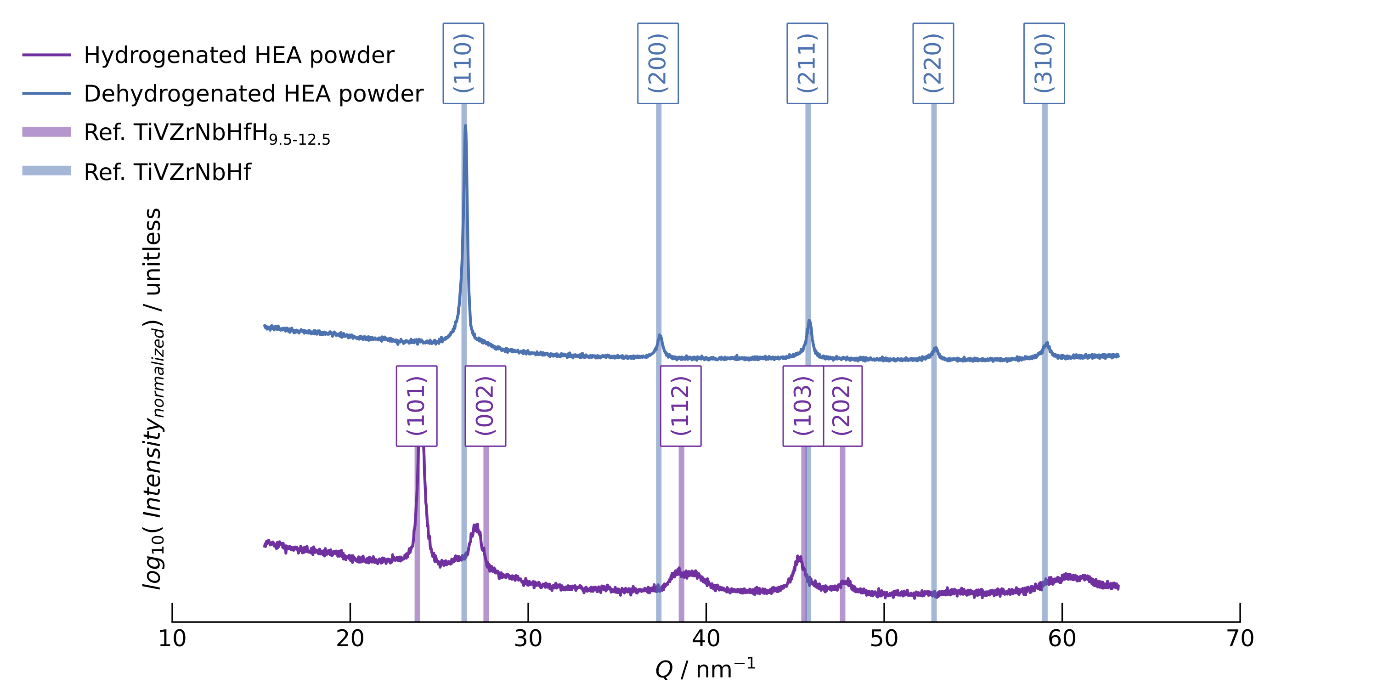
**

**Figure S15:** XRD patterns of the HEA hydride obtained by self-pulverization and the HEA powders obtained by subsequent annealing.


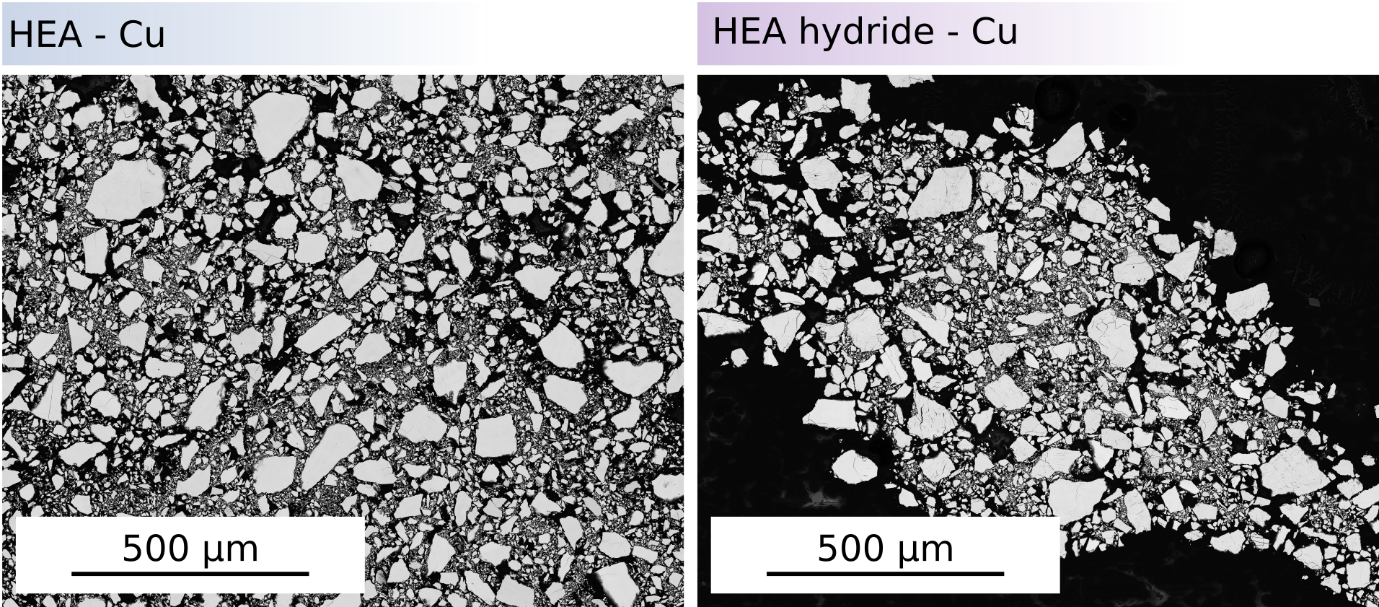


**Figure S16**: SEM micrographs of the (embedded) HEA and HEA hydride powders. The EDX results reported in Table S9 were recorded on these powders.

**Table S9:** Chemical compositions of the HEA and HEA hydride powders.

| Material | **Ti / at. %** | **V / at. %** | **Zr / at. %** | **Nb / at. %** | **Hf / at. %** |
| --- | --- | --- | --- | --- | --- |
| HEA | 22.3 ± 0.2 | 20.1 ± 0.6 | 20.2 ± 0.7 | 19.5 ± 0.9 | 17.9 ± 0.3 |
| HEA hydride | 22.3 ± 0.3 | 20.0 ± 1.0 | 20.2 ± 0.7 | 19.5 ± 1.1 | 17.9 ± 0.4 |

The hydrogen content of the hydrogenated sample was determined as 1.55 wt.% (15536 wt ppm) based on thermal desorption spectroscopy (TDS). This indicates that only minor fractions of the HEA remained un- or partially absorbed. TDS also allowed the determination of the comparable small amounts of residual hydrogen in the desorbed HEA, amounting to about 16.5 wt ppm. Consequently, the hydrogen content in the absorbed state is about 1000x larger than in the desorbed state, with the latter content in the ppm range being negligible within the scope of this study. The results of HEA hydride desorption qualitatively align with the results reported by Sahlberg et al..^[1]^ The respective TDS spectra are plotted in **Figure S17**.


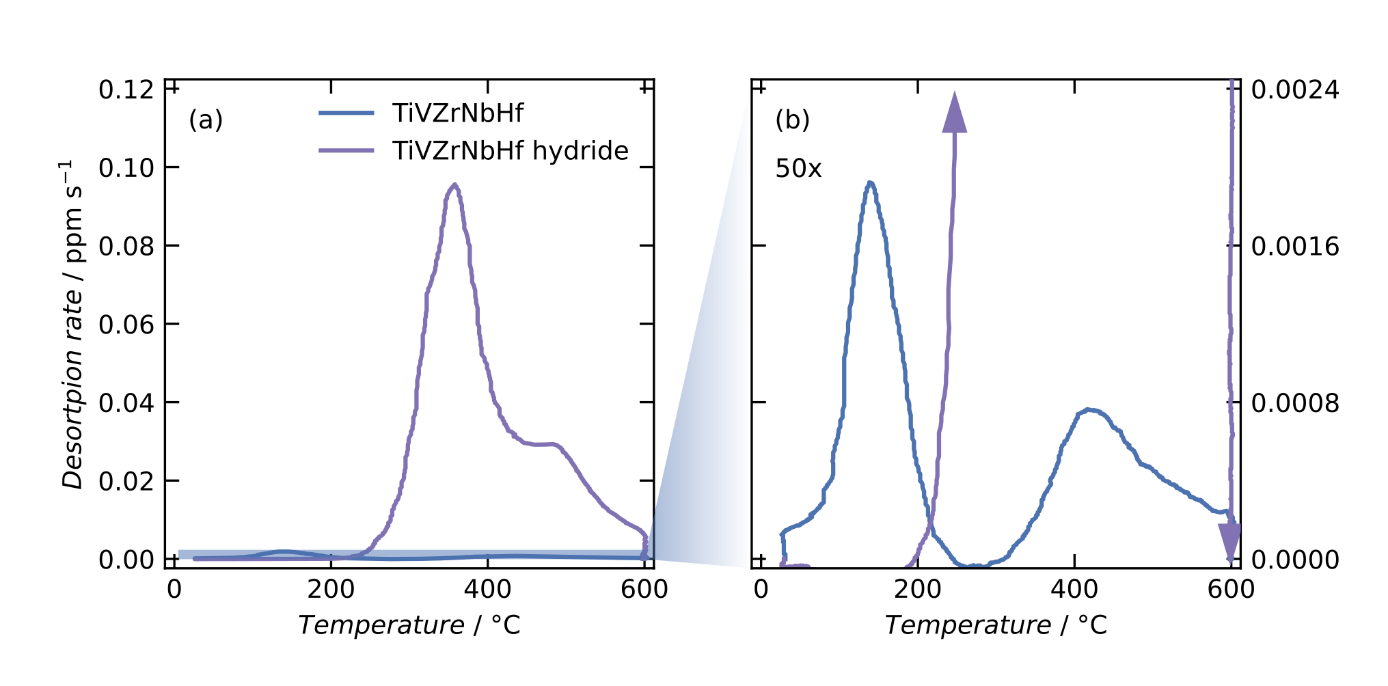


**Figure S17**: Thermal desorption spectra recorded with a heating rate of 20 K min^−1^ of (a) both the HEA and HEA hydride powders, i.e., before and after dehydrogenation at 500 °C. (b) Magnified (50x) spectra.

**References**

[1] M. Sahlberg, D. Karlsson, C. Zlotea, U. Jansson, *Sci. Rep.* **2016**, *6*, 36770.

[2] X. Yang, Y. Zhang, *Mater. Chem. Phys.* **2012**, *132*, 233.

[3] A. Takeuchi, A. Inoue, *Mater. Trans.* **2005**, *46*, 2817.

[4] R. Griessen, T. Riesterer, in *Hydrogen in Intermetallic Compounds I* (Ed.: L. Schlapbach), Springer-Verlag Berlin Heidelberg, Berlin, Heidelberg **1988**, pp. 219–284.

[5] Y. Fukai, *The Metal-Hydrogen System* (Eds.: R. Hull, J. Parisi, R. M. Osgood, H. Warlimont), 2nd ed., Vol. 21, Springer Berlin Heidelberg, Berlin, Heidelberg **2005**.

[6] D. Karlsson, G. Ek, J. Cedervall, C. Zlotea, K. T. Møller, T. C. Hansen, J. Bednarčík, M. Paskevicius, M. H. Sørby, T. R. Jensen, U. Jansson, M. Sahlberg, *Inorg. Chem.* **2018**, *57*, 2103.

[7] K. Oh-Ishi, K. Edalati, H. S. Kim, K. Hono, Z. Horita, *Acta Mater.* **2013**, *61*, 3482.

[8] A. Alhamidi, K. Edalati, H. Iwaoka, Z. Horita, *Philos. Mag.* **2014**, *94*, 876.

[9] Y. Jiang, Y. Liu, H. Zhou, S. Taheriniya, B. Bian, L. Rogal, J. T. Wang, S. Divinski, G. Wilde, *J. Mater. Sci.* **2024**, *59*, 5805.

[10] S. V. Divinski, G. Reglitz, H. Rösner, Y. Estrin, G. Wilde, *Acta Mater.* **2011**, *59*, 1974.

[11] Y. Amouyal, S. V. Divinski, Y. Estrin, E. Rabkin, *Acta Mater.* **2007**, *55*, 5968.

[12] H. Mehrer, *Diffusion in Solids* (Eds.: M. Cardona, P. Fulde, K. von Klitzing, H.-J. Queisser, R. Merlin, H. Strömer), Vol. 155, Springer Berlin Heidelberg, Berlin, Heidelberg **2007**.

[13] Y. Beygelzimer, A. Filippov, Y. Estrin, *Philos. Mag.* **2023**, *103*, 1017.

[14] N. Metropolis, A. W. Rosenbluth, M. N. Rosenbluth, A. H. Teller, E. Teller, *J. Chem. Phys.* **1953**, *21*, 1087.

[15] J. Völkl, G. Alefeld, in *Hydrogen in Metals I. Topics in Applied Physics, vol 28* (Eds.: G. Alefeld, J. Völkl), Springer Berlin Heidelberg, Berlin, Heidelberg **1978**, pp. 321–348.

[16] J. Herman, W. Usher, *J. Open Source Softw.* **2017**, *2*, 97.

[17] T. Iwanaga, W. Usher, J. Herman, *Socio-Environmental Syst. Model.* **2022**, *4*, 18155.

[18] B. B. Straumal, A. A. Mazilkin, B. Baretzky, G. Schütz, E. Rabkin, R. Z. Valiev, *Mater. Trans.* **2012**, *53*, 63.

[19] K. Edalati, S. Toh, M. Watanabe, Z. Horita, *Scr. Mater.* **2012**, *66*, 386.

[20] G. P. Grabovetskaya, I. P. Mishin, I. V. Ratochka, S. G. Psakhie, Y. R. Kolobov, *Tech. Phys. Lett.* **2008**, *34*, 136.

[21] Y. . Kolobov, G. P. Grabovetskaya, M. B. Ivanov, A. P. Zhilyaev, R. Z. Valiev, *Scr. Mater.* **2001**, *44*, 873.

[22] M. Wegner, J. Leuthold, M. Peterlechner, D. Setman, M. Zehetbauer, R. Pippan, S. V. Divinski, G. Wilde, *J. Appl. Phys.* **2013**, *114*, 183509.

[23] E. V Osinnikov, S. A. Murzinova, A. Y. Istomina, V. V Popov, A. V. Stolbovskiy, R. M. Falahutdinov, *Phys. Met. Metallogr.* **2021**, *122*, 976.

[24] N. Choi, V. Kulitckii, J. Kottke, B. Tas, J. Choe, J. H. Yu, S. Yang, J. H. Park, J. S. Lee, G. Wilde, S. V. Divinski, *J. Alloys Compd.* **2020**, *844*, 155757.

[25] K. Edalati, T. Daio, S. Lee, Z. Horita, T. Nishizaki, T. Akune, T. Nojima, T. Sasaki, *Acta Mater.* **2014**, *80*, 149.

[26] M. Kawasaki, B. Ahn, H. Lee, A. P. Zhilyaev, T. G. Langdon, *J. Mater. Res.* **2016**, *31*, 88.

[27] P. Shewmon, *Diffusion in Solids*, Springer International Publishing, Cham **2016**.

[28] J. Zhang, C. Gadelmeier, S. Sen, R. Wang, X. Zhang, Y. Zhong, U. Glatzel, B. Grabowski, G. Wilde, S. V. Divinski, *Acta Mater.* **2022**, *233*, 117970.

[29] Y. Kraftmakher, *Phys. Rep.* **1998**, *299*, 79.

[30] K. Maier, *Phys. Status Solidi* **1977**, *44*, 567.
